# A high-temperature plugging system for offshore heavy oil thermal recovery

**DOI:** 10.1371/journal.pone.0199709

**Published:** 2018-06-22

**Authors:** Yuyang Liu, Zhaoliang Li, Mao Pan

**Affiliations:** 1 School of Earth and Space Science, Peking University, Beijing, China; 2 Institute of Oil & Gas, Peking University, Beijing, China; 3 China Aero Geophysical Survey and Remote Sensing Center for Land and Resources, Beijing, China; 4 Key Laboratory of Airborne Geophysics and Remote Sensing Geology, Ministry of Land and Resources, Beijing, China; University of Akron, UNITED STATES

## Abstract

There are many heavy oil reservoirs in offshore oilfields in China. Steam and multiple thermal fluid stimulation technologies are of increasing interest and have been applied to an increasing number of projects. During the stimulation or displacement of heavy oil reservoirs during thermal recovery, several factors, such as reservoir heterogeneity, are prone to cause channeling phenomena and affect the thermal recovery effect of steam stimulation. According to the unique requirements for the stimulation of multiple thermal fluids for offshore heavy oil, this study used transmission, blocking and relieving, heat resistance and a comprehensive evaluation of parallel sand tube experiments to conduct a screening evaluation of plugging systems for the stimulation of multiple thermal fluids, screen out a commonly used plugging agent in the current stage and propose corresponding guidance for the selection basis. The results show that foam, gel, foam gel and temperature-sensitive gel systems have a good transmission performance, whereas the oil sludge exhibits a poorer performance. The phenolic resin system exhibits great plugging properties, followed by oily sludge, temperature-sensitive gel, gel, foam gel and foam. Considering about washing resistance properties, phenolic resin system shows the best quality, followed by oily sludge and temperature-sensitive gel. The oily sludge system brings the best performance in plugging a high-permeability channel than phenolic resin gel and temperature-sensitive gel.

## Introduction

The offshore oil and gas fields in China are mainly distributed throughout Bohai Bay, the East China Sea, and the west and east areas of the South China Sea. Currently, more than 20 heavy oil fields have been discovered in the Bohai Sea area[1]. The reserve discovery and construction capacity of heavy oil in the Bohai Sea area are particularly important[2–6]. Compared to an onshore heavy oil field, an offshore heavy oil field reservoir is relatively deep and constrained by the space of the offshore platform, thus making it difficult to display the equipment[7–10]. Furthermore, onshore heavy oil exploitation cannot be fully applied to the sea due to economic factors. To promote the application and development of thermal recovery technology for the exploitation of offshore heavy oil fields in China, we conducted research on thermal recovery stimulation technology for multiple thermal fluids of offshore heavy oil at an oilfield from 2008 to 2010 and observed a notable increase in yield. We also discovered significant fluid channeling phenomena of steam and multiple other thermal fluids after several rounds of stimulating the thermal fluids, which severely affects subsequent stage development.

The Bohai Sea heavy oil reservoirs are generally distributed in unconsolidated sandstone formations consisting of fluvial facies or fluvial-delta facies deposits, where the reservoir cementation is loose and has large pores, high permeability and significant heterogeneity. During thermal exploitation, after many rounds of stimulating the recovery of oil wells, the contradiction between the layers and the planes became increasingly prominent due to the effects of steam ultra-overburden, plane fingering, reservoir heterogeneity and other factors, in turn causing the steam difference between the high- and low-permeability layers. The high-permeability layer is a strong steam layer, whereas the low-permeability layer is a weak steam layer that does not even suck steam. Steam channeling may also be produced in the high rounds of the stimulation stages, which causes a disturbance in the steam channeling between wells. Steam flooding inevitably aggravates this trend.

When oilfield development enters a high water-cut period, the heterogeneity and sensitivity of the reservoir leads to an increase in the size of the pore throat, and the injected water forms a channeling duct along the high-permeability zone, which severely influences the development of oilfield[11, 12]. The formation mechanism and the identification and characterization of the channeling duct are of great significance, and many scholars have accordingly performed numerical computation and physical simulation research on these topics[13–18].

Several scholars have conducted relative research on steam and the stimulation of multiple thermal fluids and fluid channeling: Jiang Jie (2014) analyzed the channeling characteristics of multiple thermal fluids in horizontal wells according to a reservoir’s physical properties, fluid viscosity and production dynamics. The sensitivity of the factors influencing channeling were quantitatively studied via numerical simulation, and the key influencing factors were screened out by introducing the “coefficient of variation method.”[19] Jiang Jie (2015) also used the gray relational method to analyze the influences of various factors on the channeling of multiple thermal fluids and quantified their effects and relationship by calculating the correlation of each factor[20]. Wu Haijun (2015), considering the problem of gas channeling between horizontal wells under large duct conditions during the channeling of multiple thermal fluids, proposed and used the flow coefficient, channeling distance and shape factor as parameters in the numerical simulation method to quantitatively characterize the size of the gas channeling and to discuss the influences of large duct permeability and injection volume on the three parameters[21].

The most direct and effective way to solve the problem of uneven steam on vertical and horizontal planes is to apply high-temperature plugging and profile control technology. This approach improves the steam injection profile and the utilization and recovery of heavy oil reservoirs by solving the problem of uneven steam on vertical and horizontal planes[22–25].

Currently, the relevant research at home and abroad is focused on the development and test evaluation of certain plugging agents. There is no systematic, comprehensive evaluation of various plugging systems or corresponding guidance for the selection basis.

Therefore, according to the gap in the existing research, this paper first introduces the technical requirements of the plugging system for thermal recovery of offshore heavy oil and then selects six different types of plugging systems, namely, gel, foam, foam gel, thermo-sensitive gel, thermosetting gel and oily sludge. Finally, according to the characteristics of the heavy oil reservoirs in the Bohai Sea, the various plugging agents of this paper are comprehensively evaluated and compared through transmission, plugging and parallel sand pipe experiments, and corresponding guidance for the selection basis is provided.

## Technical characteristics of the thermal recovery of offshore heavy oil

For the thermal recovery of offshore heavy oil, if the temperature of the injected multiple thermal fluids (steam, CO_2_ and N_2_) is up to 300°C, with multiple rounds of steam stimulation, an alternating temperature field will be produced in the reservoir, as CO_2_, N_2_ and other gases accompany the steam injection. Therefore, in addition to needing a plugging agent with a good plugging effect at high temperatures, CO_2_, N_2_ and other gases must have a good plugging effect when plugging the steam channeling. According to the physical parameters and steam injection process of heavy oil reservoirs, the plugging system, which can be used for heavy oil thermal recovery wells, should have the following characteristics:

1. Lower initial viscosity and a good injection quality;
2. Suitable and adjustable gelation(precipitation) time, typically between 8 and 50 h;
3. Good temperature resistance of more than 200°C;
4. Good compatibility performance with formation rocks and liquid (mainly includes oil and water) and salt resistance;
5. Good plugging ability and long-term stability, easy removal, and little pollution to layers;
6. Extensive source of raw materials, with low prices and economic viability.

Offshore oil and gas exploitation platforms have certain limitations, such as the volume and power limitations of pumps and water treatment equipment, high environmental protection requirements, and a high-temperature plugging system output fluid that does not affect the platform process. Besides the aforementioned properties, the development of the offshore plugging system should also have following characteristics in comparison with those of onshore ones.

1. Easy to configure;
2. Small impact on the pipe column and platform process;
3. Good pump performance;
4. Technology maturity of the plugging system.

To solve the problem of the steam channeling of heavy oil in an offshore oilfield, this study first comprehensively evaluates the current plugging agents with high temperature resistance. The analysis is summarized in Table 1.

**Table 1:** Evaluation and comparison of various types of profile control agents and plugging agents.

Agent type	Advantages	Disadvantages	Applicability to offshore thermal recovery
High-strength compound plugging agent	High plugging intensity	Poor compatibility with formation rocks and liquid, certain operating risks	Not applicable
Precipitation-type profile control agent	Low cost, good stability and selectivity, anti-shearing	Precipitation is greatly affected by temperature and pressure (not adjustable), lower precipitation efficiency, larger damage to layers (Poor compatibility with formation rocks and liquid)	Not applicable
Foam plugging agent	Preferential access to high water large ducts, selectivity, the Jamin effect, reduction of the water-oil interfacial tension	Steam affects the foaming effects, and oil saturation has a considerable effect on foam profile control	Applicable
Temperature-sensitive gel	High pump performance, low viscosity at low temperatures, high viscosity at high temperatures, releasable	Difficult for research and development	Applicable
Microsphere plugging agent	In-depth profile control, removable profile control	Strict requirements of the microsphere properties, a higher cost, and technology is not mature	Not applicable
Oily sludge	Good compatibility performance, little damage to layers, energy conservation and environmental protection	Oily sludge pretreatment is required	Applicable
Cement plugging agent	High strength, low cost, suitable for various temperatures	Permanent plugging (not easy to removal) and high construction risk	Not applicable
Improved inorganic granule plugging agent	Good performance of high and super-high-permeability plugging	Easy precipitation (not adjustable), low selectivity, intensity is not too high,	Not applicable
Resin plugging agent	High plugging intensity, high temperature resistance	Small dealing radius	Applicable

Considering the maturity of the system and the domestic field applications, this paper chose the following plugging systems to conduct experimental evaluation research: foam, gel, foam gel, temperature-sensitive gel, thermosetting resin plugging agent and oily sludge. The common high temperature resistant formulations after the optimization of each system are used. For the gel system, a polymer concentration of 4% and a crosslinking agent concentration of 0.05% combined with other additives in proportion were used. The foam system comprised a polymer concentration of 1000 ppm, a high-temperature foaming agent concentration of 0.4–0.5%, and a gas-water ratio of 3:2 (5 MPa, 50°C). The foam gel system consisted of a polymer concentration of 4‰, a crosslinking agent concentration of 0.05%, other additives in proportion, a high-temperature foaming agent concentration of 0.3%, and a gas-water ratio of 100. For the temperature-sensitive gel system, temperature-sensitive gel powder was directly configured to a 1% temperature-sensitive gel solution. For the phenolic resin system, a phenolic resin prepolymer was synthesized by a two-step alkali catalytic method. The oily sludge system comprised a polymer concentration of 1%, an oily sludge solid content of 5%, and an oily sludge particle diameter ratio, according to the “ideal filling” theory proposed by Hands et al., that is, the ideal temporary plugging effect can be achieved when the d_90_ value (meaning that 90% of the particle diameter is smaller than this value) of the temporary plugging agent on its particle diameter cumulative distribution curve is equal to the maximum hole throat diameter of the layers.

Transmission, plugging, and temperature resistance are three important indicators to measure the performance of a high-temperature plugging system for channeling. Transmission in this case means the degree of difficulty of injecting the plugging agent into the layers, mainly by characterizing the pressure change in the injection process. Plugging is the effect of plugging agents on the layer plugging, mainly through the characterization of the resistance factor, residual resistance factor and plugging rate. Finally, the temperature resistance is the effect of plugging agents on the layer plugging at a high temperature, mainly through the characterization of the breakthrough pressure gradient and plugging rate.

Based on the characteristics of heavy oil reservoirs in the Bohai Sea area, a transverse experimental comparison is performed and the performance indices of the above common plugging agents are evaluated to determine the applicable conditions and selection basis of various plugging agents.

## Transmission of a high-temperature channeling plugging system

**Fig 1:**
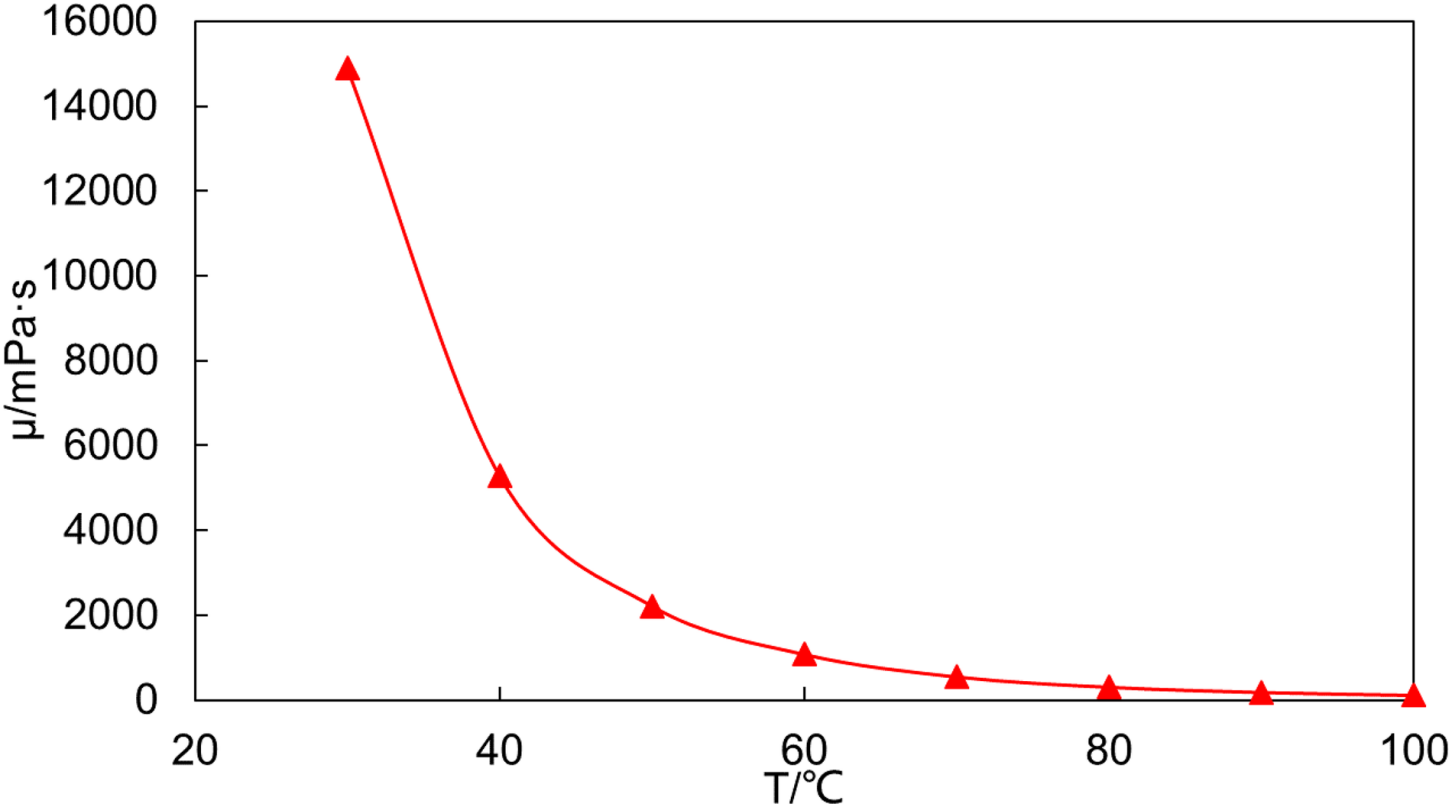
Viscosity-temperature curve of the dehydrated crude oil from a heavy oil reservoir in the Bohai Sea area.

The crude oil for the experiment is the dehydrated crude oil from a heavy oil reservoir in the Bohai Sea area, and the viscosity-temperature characteristics are as shown in Fig 1.

**Fig 2:**
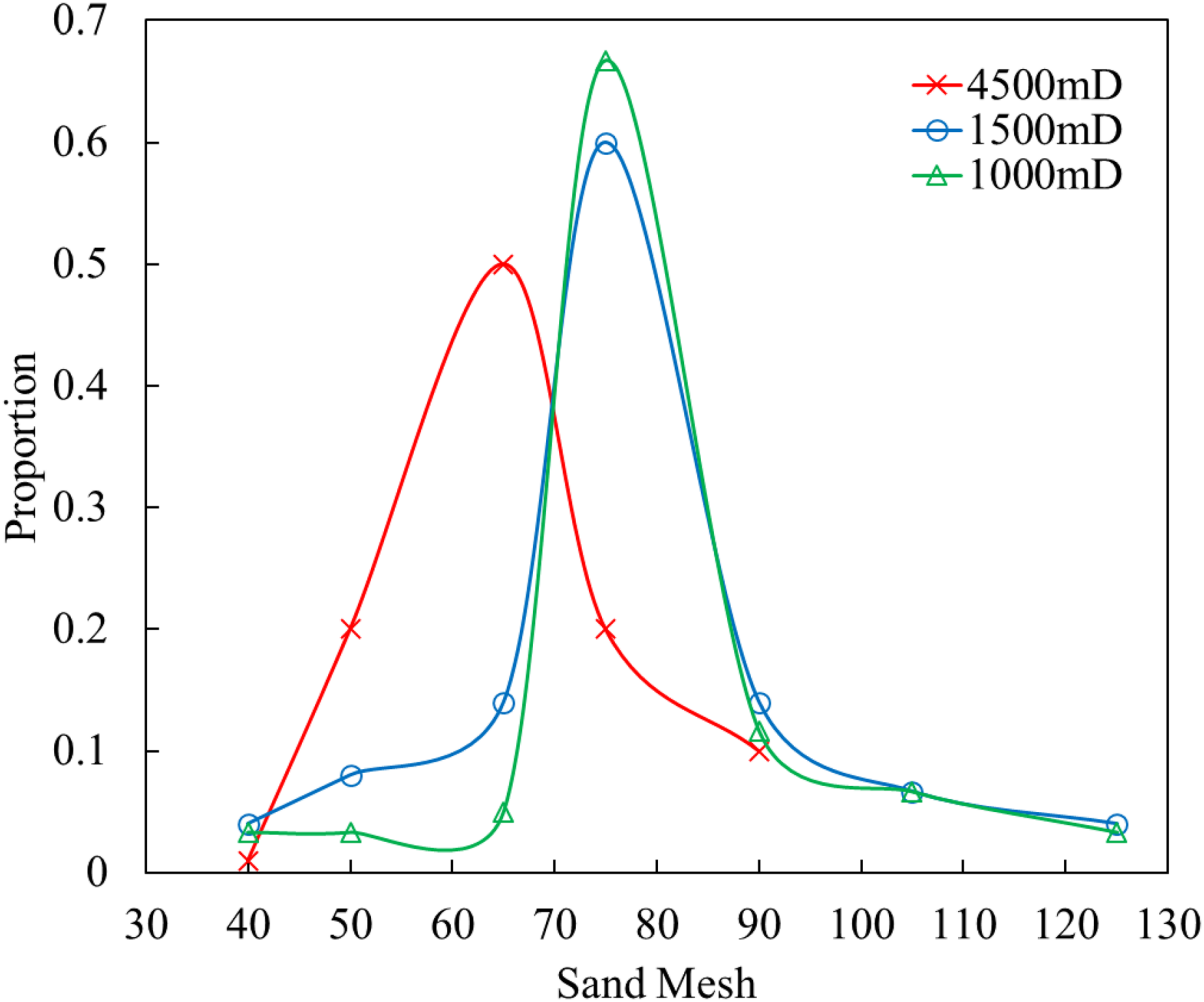
Sand particle diameter distribution curves of artificial cores with different permeabilities.

According to the actual sand particle diameter ratio of the Bohai area, the configuring permeability is artificial cores of 1,000 mD, 1,500 mD, and 4,500 mD. The particle diameter ratio is shown in Fig 2.

The injection pressure of the plugging system changes with the injection quantity. A good injection performance ensures that the pressure will not rise sharply during the injection process, thus guaranteeing safety and a smooth construction process. In this experiment, the high-permeability core of 4,500 mD is selected to better determine the plugging effect of various plugging agents.

A sand filling tube with a diameter of 2.5 cm and a length of 15 cm is used in the experiment. The residual resistance factor and the plugging rate are obtained by recording the breakthrough pressure of steam flooding as follows:

1. Use the pump with constant speed and pressure to inject water at 2 mL/min, test the permeability, and record the pressure difference;
2. Continuously inject 1 pore volume (PV) plugging agents with different gas-liquid ratios at 2 mL/min, record the pressure difference between the two ends of the core pipe, and calculate the resistance factor;
3. Test the water permeability after plugging, following the displacement with steam temperatures of 100, 200, and 300°C, and record the pressure changes during the displacement process.

**Fig 3:**
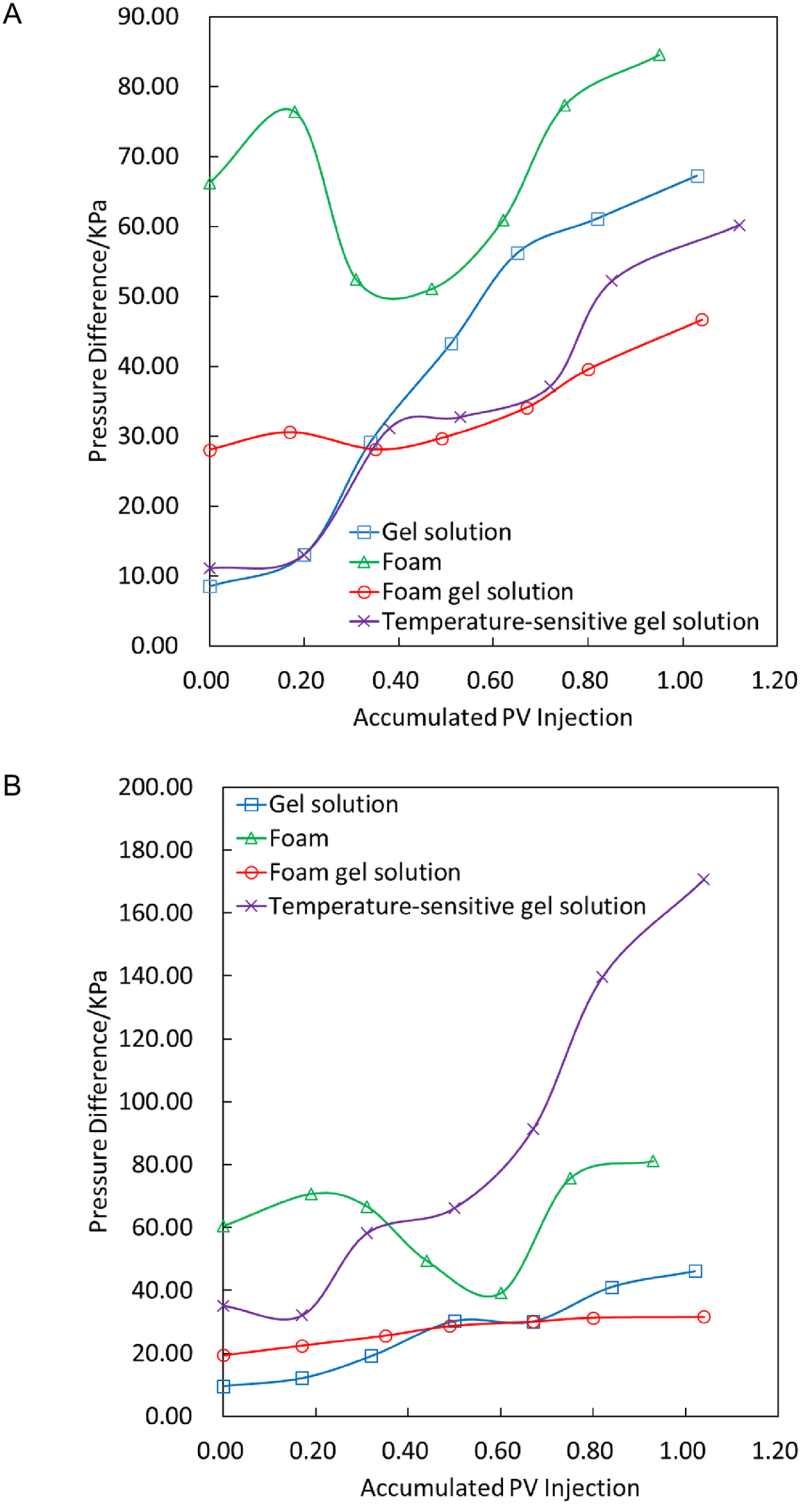
Transmission experiments of different plugging systems at 50°C and 80°C. (A) 50°C. (B) 80°C.

As shown in Fig 3, the transmission performances of foam, gel and foam gel are better under 80°C than under 50°C. The foam leads to fluctuations in the injection pressure during the injection process due to its unstable system. The transmission performances of the temperature-sensitive gel system and phenolic resin system worsen with increasing temperature, which coincides with the temperature sensitivity and thermoset of the systems. The injection pressure does not exhibit a sudden increase with a slow increase in the injection volume, which indicates a good transmission performance. The transmission performance of the oily sludge system is poor, and the solid phase causes the pressure to present larger irregular fluctuations in the transmission process. The viscosity of the phenolic resin solution before curing is similar to the viscosity of water. The phenolic resin solution has less of a pressure difference in the transmission process and exhibits a good transmission performance. The pressure change is not obvious and is thus not shown in Fig 3.

## Study on plugging the high-temperature channeling plugging system

Plugging performance is the most important performance of the plugging system and is directly related to the practical application effect of the plugging system. The residual resistance factor and plugging rate are used to evaluate the plugging performance of the plugging agents by injecting different types of plugging agents into the sand filling pipe model (after gelation) at different temperatures, recording the pressure difference through the water drive, and calculating the breakthrough pressure gradient and resistance factor.

The breakthrough pressure, which reflects the plugging strength and is the maximum displacement pressure in the displacement process, is an important parameter for measuring the ability of the gel to plug. The breakthrough pressure gradient can be calculated as follows:

$$P_{t}=\frac{P_{\text{max}}}{L}$$ (1)

In this formula, *P*_*t*_ is the breakthrough pressure gradient, *P*_*max*_ is the breakthrough pressure, and *L* is the length of the sand filling pipe. The calculations of the plugging rate and resistance factors after plugging use the breakthrough pressure *P*_*max*_.

Washing resistance is different from plugging performance but they share a certain relationship, that is, a better washing resistance corresponds to a higher plugging performance that the gel can retain after breaking through. Thus, the gel cannot be easily flushed away by the subsequently injected displacing fluids, which means that the effect of the permeability decline can continue for a longer period of time. Washing resistance is often represented by a residual resistance factor *F*_rrw_ and the plugging rate after breakthrough with a value of *η*.

The residual resistance factor is the ratio of the penetration rate before and after plugging, which can be converted into the pressure gradient, i.e., the ratio of the pressure gradient after and before plugging. The residual resistance factor is calculated by formula (2):

$$F_{\text{rrw}}=\frac{K_{\text{wa}}}{K_{\text{wb}}}$$ (2)

The plugging rate is the ratio between the differences of the penetration rate before and after plugging and the penetration rate before plugging, which can be calculated using formula (3):

$$\eta =\frac{K_{\text{wa}}-K_{\text{wb}}}{K_{\text{wa}}}\times 100\%$$ (3)

In this formula, *F*_rrw_ is the residual resistance factor, *K*_wa_ is the water test permeability before plugging, *K*_wb_ is the water test permeability after plugging, and *η* is the plugging rate.

The residual resistance factors after steam flooding and the plugging rate after steam flooding are been calculated using the stable pressure after steam flooding.

**Fig 4:**
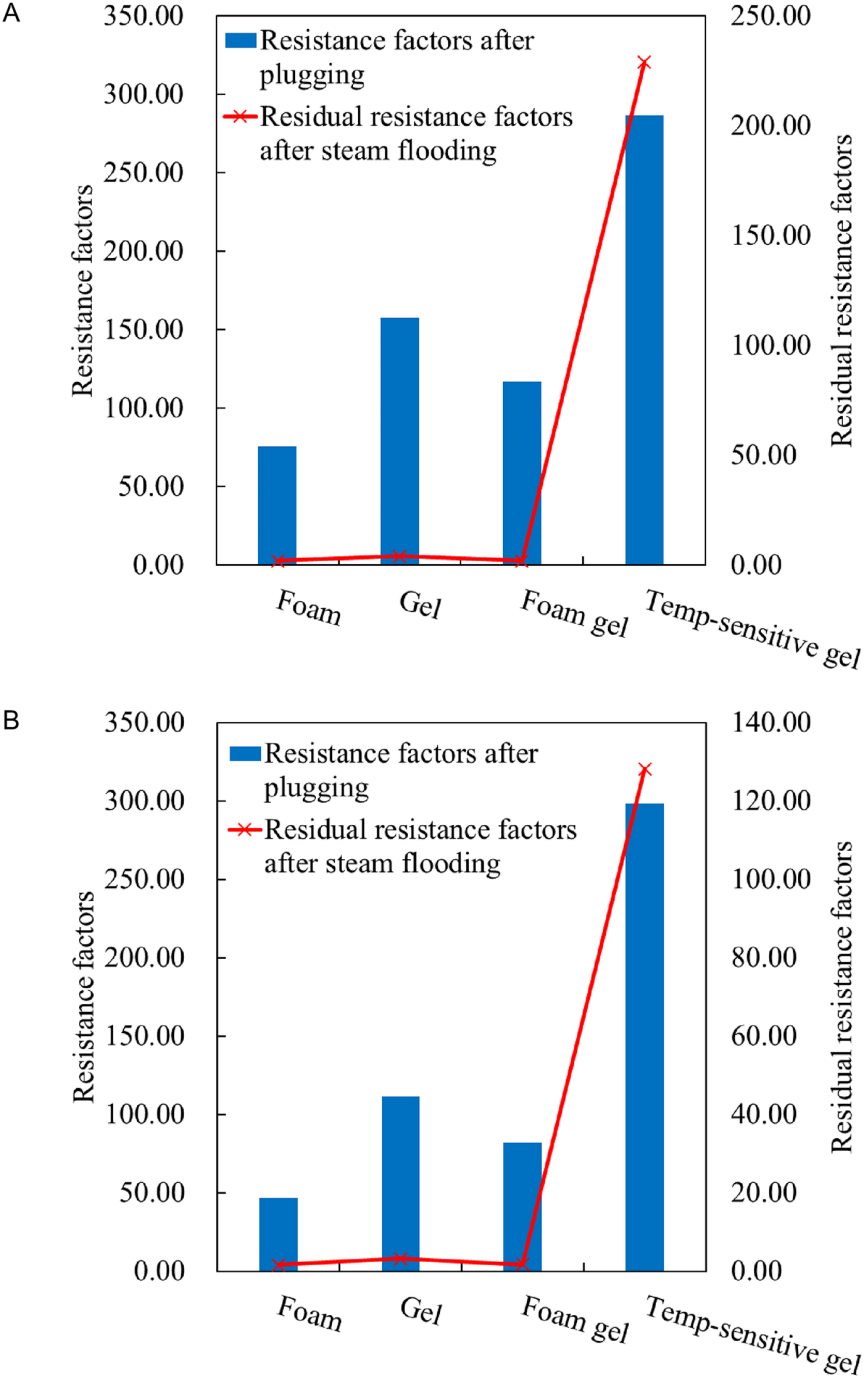
Resistance factors after plugging and residual resistance factors after steam flooding of different plugging agents at 100°C and 200°C. (A) 100°C. (B) 200°C.

As shown in Table 2 and Fig 4, the resistance factor of each plugging agent after plugging is between 75.58 and 298.28, and the plugging rate is more than 97%. Therefore, each plugging system can achieve a good plugging effect under high-temperature conditions in a short time.

**Table 2:** Evaluation experiments of the plugging performance of a high-temperature channeling plugging system.

Plugging agents	Temperature (°C)	Resistance factor after plugging	Residual resistance factor after steam flooding	Plugging rate
Foam	100.00	75.58	2.01	0.9867
	200.00	46.85	1.59	0.9767
Gel	100.00	157.52	4.14	0.9957
	200.00	111.27	3.19	0.9910
Foam gel	100.00	117.35	1.97	0.9851
	200.00	82.07	1.69	0.9961
Temperature-sensitive gel	100.00	286.50	229.14	0.9962
	200.00	298.28	128.21	0.9922

However, Table 2 illustrates that after injecting high-temperature steam for 10 minutes, the plugging effects of foam, gel and foam gel decrease significantly, with the residual resistance factors dropping to 1–3, at which time the plugging effects of foam, gel, and foam gel are poor.

The temperature-sensitive gel, oily sludge and phenolic resin system have a good plugging effect at high temperatures. Thus, washing resistance experiments for the temperature-sensitive gel, oily sludge and phenolic resin system are performed to evaluate the high-temperature stability of the three systems. Phenolic resin plugging agents are commonly used as high-temperature resistant materials, as the temperature resistance of these materials can reach 500°C after curing. Therefore, it is sufficient to study the washing resistance of phenolic resin at 300°C. The results of the experiment are shown in Table 3.

**Table 3:** Evaluation results of the washing resistance of different plugging systems.

Plugging agents	Temperature (°C)	Resistance factors after plugging	Residual resistance factors after steam flooding	Plugging rate after steam flooding
Temperature-sensitive gel	100	286.50	229.14	0.9956
	200	298.28	128.21	0.9922
	300	220.08	39.5	0.9746
Oily sludge	100	6510.49	1659.26	0.9993
	200	3219.35	1175.48	0.9991
	300	2529.48	168.79	0.9940
Phenolic resin	300	5799.70	304.34	0.9967

**Fig 5:**
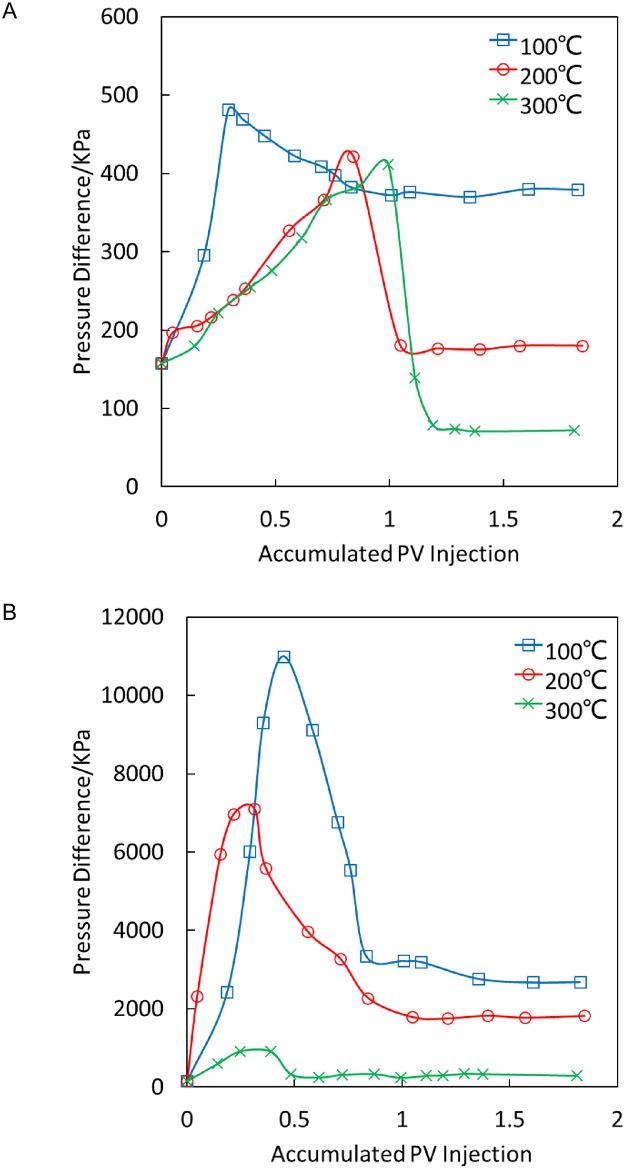
Relations between the steam flooding pressure difference and accumulated injection volume of temperature-sensitive gels and oily sludge after plugging at different temperatures. (A) Temperature-sensitive gels. (B) Oily sludge.

Fig 5(A) illustrates that for temperature-sensitive gel plugging, the breakthrough pressure gradient in the process of steam flooding at 100°C is approximately 4.13 MPa/m, which is slightly decreased at 200°C and 300°C. However, with increased steam injection, the stable pressure and breakthrough pressure are highly similar at 100°C, whereas the stable pressure drops considerably at 200°C and 300°C. The stable water test permeability indicates that the plugging rates after steam flooding are 99.22% and 97.46% at 200°C and 300°C, respectively, which means that the temperature-sensitive gel maintains a good plugging effect.

Fig 5(B) illustrates that for oily sludge plugging, the breakthrough pressure difference differs considerably at different temperatures in the process of steam flooding, reaching as high as 90 MPa/m and 57.9 MPa/m at 100°C and 200°C, respectively, whereas the breakthrough pressure difference is only approximately 8.26 MPa/m at 300°C. As the temperature rises, the breakthrough pressure difference changes considerably because the polymer solution used in the suspended oily sludge has been destroyed with the increasing temperature, which causes the stability of the oily sludge system to worsen, in turn, partially destroying the sludge plugging and resulting in a reduced plugging pressure difference. After the breakthrough, the stable pressure difference drops even further. The water test permeability after the pressure difference becomes stable illustrates that the plugging rates after steam flooding at 100°C, 200°C and 300°C are 99.93%, 99.91% and 99.4%, respectively, indicating that the plugging effect remains good.

**Fig 6:**
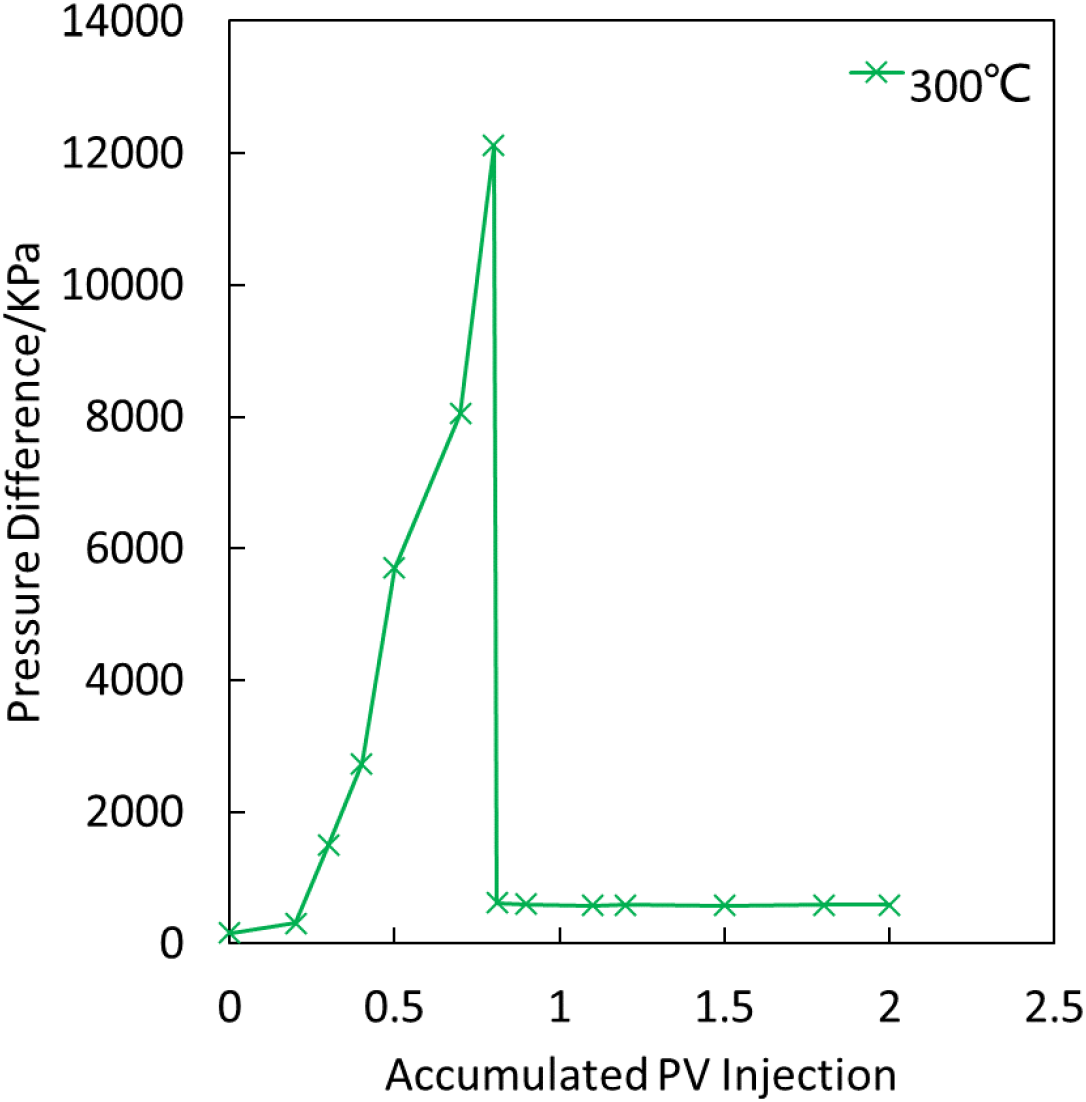
Relations between the steam flooding pressure difference and accumulated injection volume of phenolic resin after plugging at 300°C.

Fig 6 illustrates that for phenolic resin plugging, when steam injection occurs at 300°C, the breakthrough pressure gradient is up to 100 MPa/m due to the rapid increase in the plugging pressure to the breakthrough pressure, which decreases dramatically after the breakthrough and gradually stabilizes with the steam flooding. After steam flooding at 300°C, the plugging rate of the phenolic resin system reaches 99.67%.

## Experimental study on parallel sand pipes

Because of the stratum heterogeneity and the differences in the fluidity between the steam and reservoir fluids, steam (gas) channeling often occurs during reservoir development, which restricts the possible enhancement in recovery.

**Fig 7:**
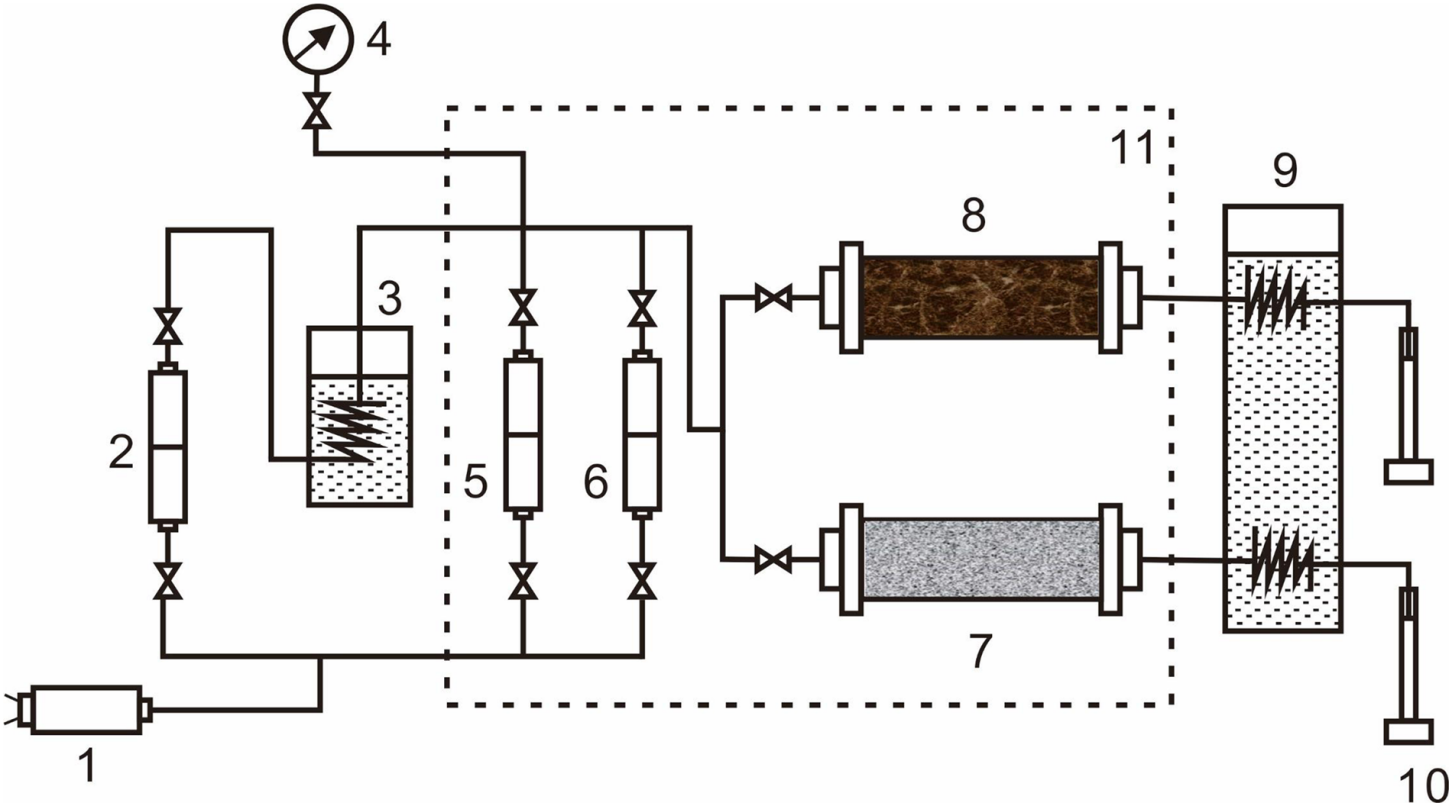
Flow diagram of a double-tube parallel plugging simulation. 1-constant flow pump; 2-water containers; 3-steam generators; 4-pressure sensors; 5-oil containers; 6-plugging system containers; 7, 8-sand filling pipes; 9-cooling water bath; 10-measuring cylinder; 11-thermostat.

This experiment evaluates the selective plugging ability of different high-temperature plugging systems by simulating the stratum heterogeneity using different permeability levels of the parallel sand filling pipes to improve the effectiveness of the thermal recovery. The experimental installation process is shown in Fig 7.

According to the particle diameter ratio of formation sands, sands with different mesh numbers are obtained after screening, and the layers of different permeability levels are simulated by filling sand samples with particles of different diameters. After saturating the formation water and heavy oil, the filling model with the necessary saturation (φ 25 mm×150 mm) was obtained using steam flooding. In the foam plugging experiment, a certain quantity of vapor is injected into two sand filling models after the foam is injected. In the plugging experiments of foam gel and gel, after the injection is complete, the gelation must maintain a constant temperature and pressure for a certain period of time. Then, a certain quantity of steam flooding is injected into the two sand filling models. The injection pressure changes of the water injection, oil injection, foam gel injection and steam injection are recorded in detail.

The main experimental steps are as follows. First, use the pump with a constant speed and pressure to inject water at 2 mL/min, test the permeability, and record the pressure difference. Second, use the pump with a constant speed and pressure to inject saturated oil at a speed of 1 mL/min and record the stable pressure and the quantity of saturated oil. Third, inject steam at 200°C and 300°C at a speed of 2.0 mL/min until the comprehensive water content reaches 98%. At this time, record the oil and water production of the two sand pipes and inject the plugging agents into the parallel sand pipes (the gel will be placed in a thermostat of 56°C for 12 h, waiting for gelation). Finally, continuously inject steam at a speed of 2 mL/min and record the water yield of the two sand pipes in the subsequent steam flooding.

In the analysis of the experimental results of parallel sand pipes, the final recovery rate of foam system, the foam gel system is the same as that of the gel system with an increasing steam injection volume. Therefore, the following parallel experimental results and conclusions do not describe the foam and foam gel.

### Parallel experimental study of different plugging system

The condition is as follows: gel plugging, 1,000/1,500 mD, parallel experimental results at 200°C and 300°C, with an injection volume of 1 PV.

**Fig 8:**
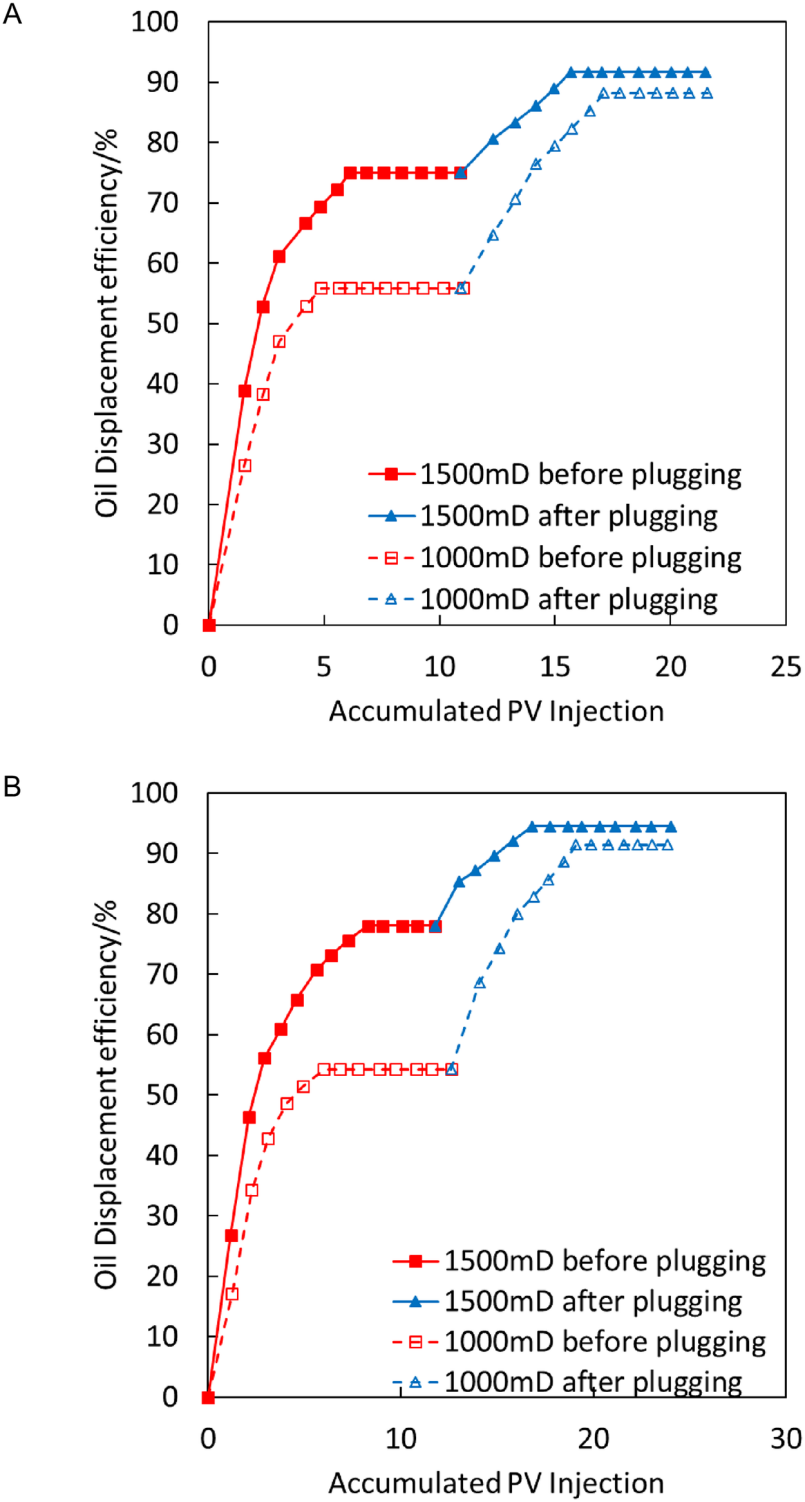
Comparison of oil displacement efficiency before and after gel plugging at 200°C and 300°C. (A) 200°C. (B) 300°C.

Fig 8 illustrates that after gel plugging, the recovery rate of low-permeability sand pipes is increased by 32.35% in the displacement process at a high temperature of 200°C, and the recovery rate of low-permeability sand pipes is increased by 37.14% in the displacement process at 300°C. Thus, the plugging effect of steam flooding at 300°C is good, thus effectively improving the suction capacity of low-permeability sand pipes.

The condition is as follows: temperature-sensitive gel plugging, 1,000/1,500 mD, parallel experimental results at 200°C and 300°C, with an injection volume of 1 PV.

**Fig 9:**
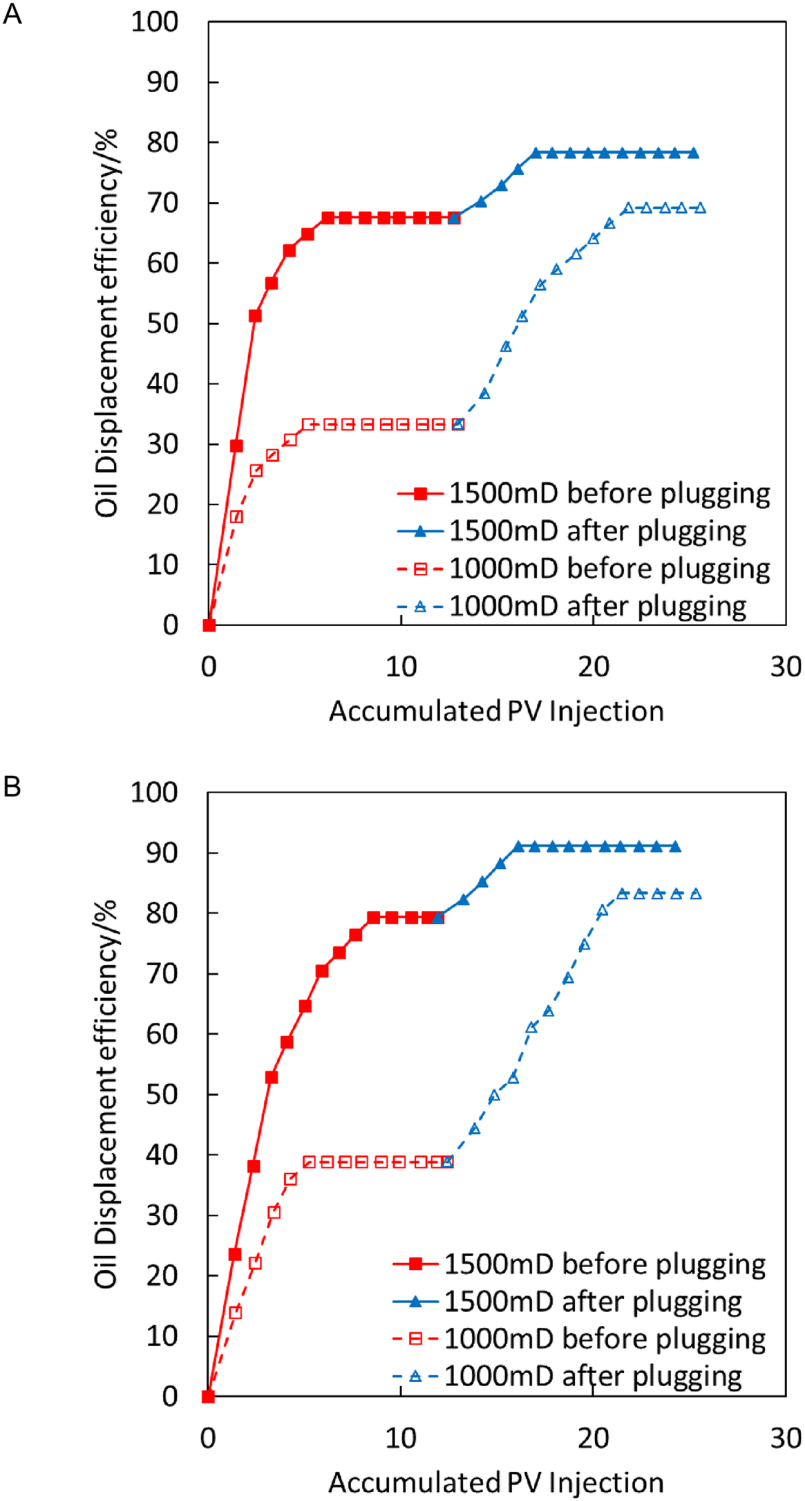
Comparison of oil displacement efficiency before and after temperature-sensitive gel plugging at 200°C and 300°C. (A) 200°C. (B) 300°C.

Fig 9 illustrates that the recovery rate of low-permeability sand pipes is increased by 35.9% with the high-temperature displacement process at 200°C after temperature-sensitive gel plugging, whereas the recovery rate of high-permeability sand pipes is increased by 10.81%. The recovery rate of low-permeability sand pipes is increased by 44.44% in the displacement process at 300°C after plugging, whereas the recovery rate of high-permeability sand pipes is increased by 11.73%. Thus, in the process of steam flooding at 200°C and 300°C, the recovery rate of low-permeability sand pipes is considerably higher after plugging, demonstrating a good plugging performance. The recovery rate of a low-permeability sand pipe at 300°C steam flooding is increased by 44.44%. The recovery rate of the low-permeability sand pipe is 35.9% higher at 300°C steam flooding than that at 200°C steam flooding after plugging, indicating that the temperature-sensitive gel has a better plugging effect at 300°C steam flooding; this finding is consistent with the results for the thermal thickening performance of the temperature-sensitive gel.

The condition is as follows: oily sludge plugging, 1,000/1,500 mD, parallel experimental results at 200°C and 300°C, with an injection volume of 1 PV.

**Fig 10:**
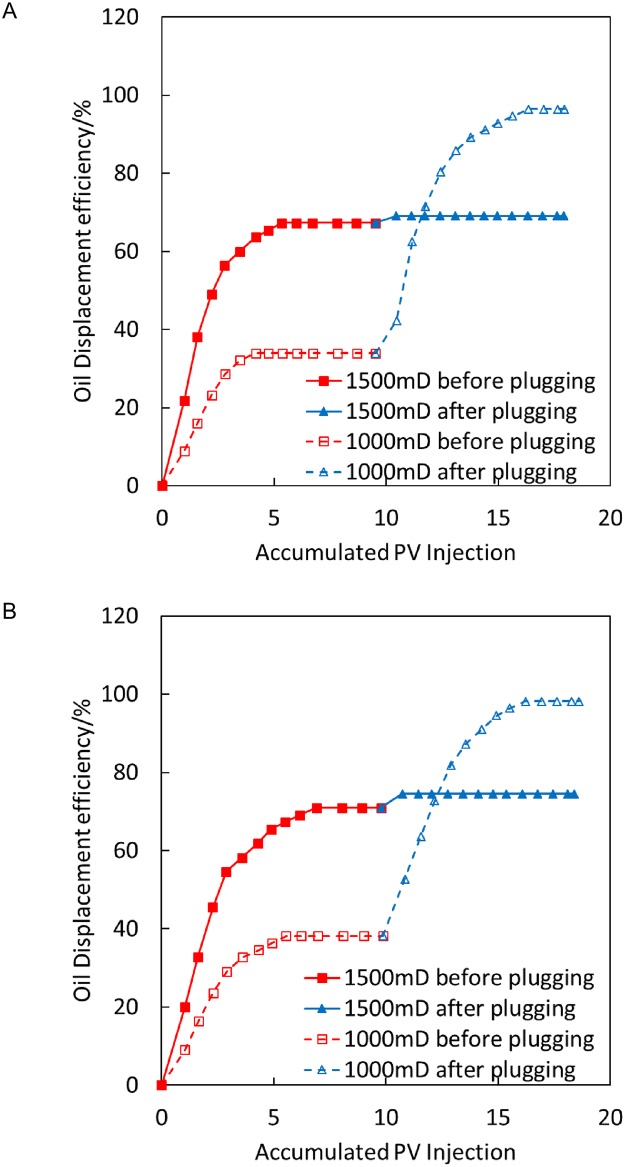
Comparison of oil displacement efficiency before and after oily sludge plugging at 200°C and 300°C. (A) 200°C. (B) 300°C.

Fig 10 illustrates that the recovery rate of low-permeability sand pipes in the high-temperature displacement process at 200°C is increased by 59.5% after oily sludge plugging, whereas the recovery rate of high-permeability sand pipes is increased by 1.82%. The recovery rate of low-permeability sand pipes in the displacement at 300°C is increased by 60% after plugging, whereas the recovery rate of high-permeability sand pipes is increased by 3.64%. Thus, the seepage capability of the high-permeability channel is greatly decreased after oily sludge plugging, which makes the recovery rate of low-permeability sand pipes considerably higher. The plugging result of oily sludge is better under temperatures of 200°C and 300°C.

The condition is as follows: phenolic resin plugging, 1,000/1,500 mD, parallel experimental results at 200°C and 300°C, with an injection volume of 0.25 PV.

**Fig 11:**
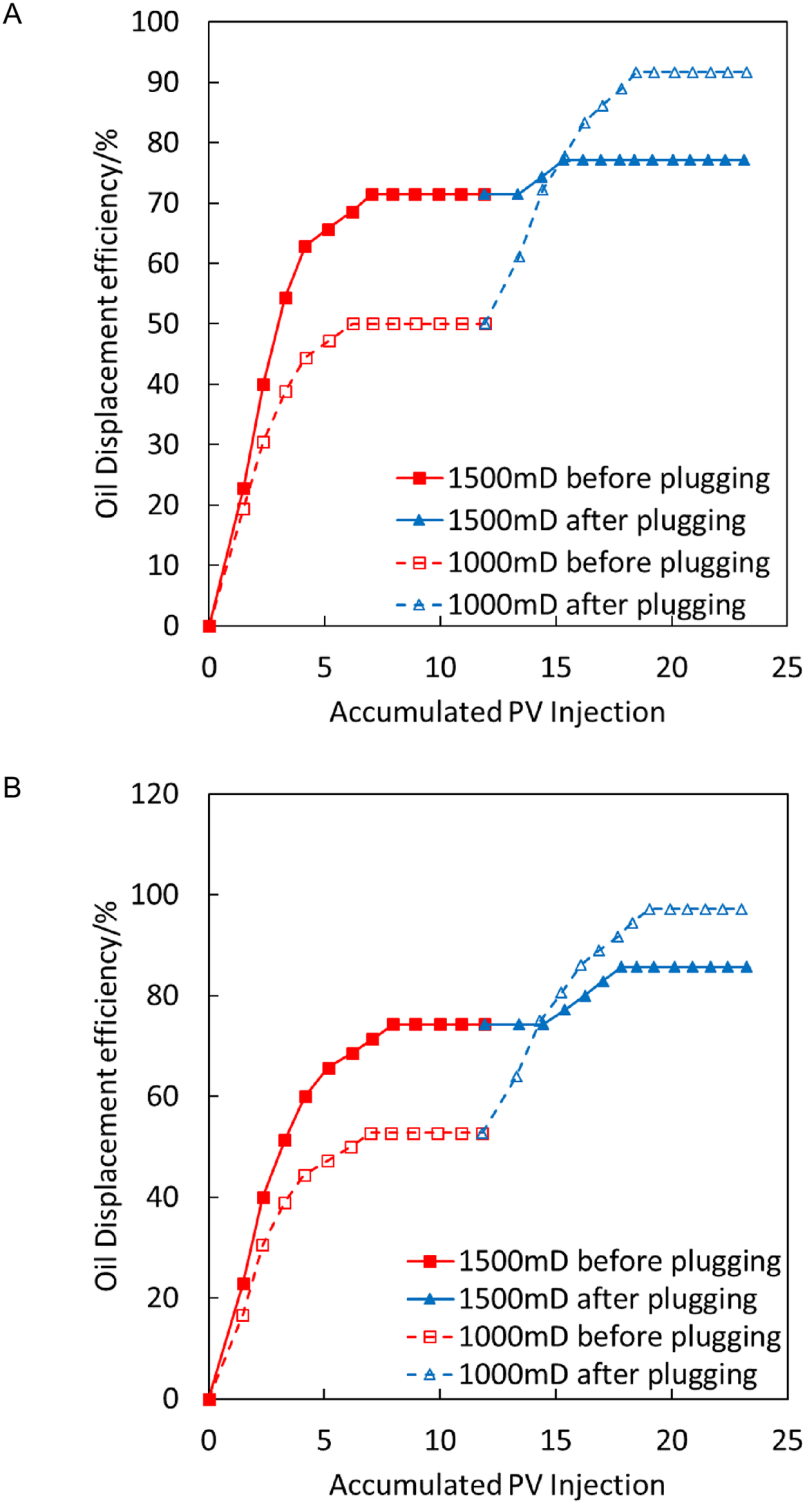
Comparison of oil displacement efficiency before and after phenolic resin plugging at 200°C and 300°C. (A) 200°C. (B) 300°C.

Fig 11 illustrates that the recovery rate of low-permeability sand pipes in the high-temperature displacement process at 200°C is increased by 41.67% after phenolic resin plugging, whereas the recovery rate of high-permeability sand pipes is increased by 5.71%. The recovery rate of low-permeability sand pipes in the displacement process at 300°C is increased by 46.44% after plugging, whereas the recovery rate of high-permeability sand pipes is increased by 11.43%. Thus, the percolation capacity of the high-permeability channel decreases considerably after phenolic resin plugging, and the recovery rate of low-permeability sand pipes increases considerably. Therefore, phenolic resin plugging can achieve a good plugging effect at 200°C and 300°C.

## Conclusion

Considering the characteristics of heavy oil reservoirs in the Bohai Sea area, this paper comprehensively evaluates and compares the various common plugging agents to guide the selection of a plugging system onsite through experiments on transmission, plugging and parallel sand pipes. The following conclusions can be drawn from this study:

1. The evaluation of the transmission experiment indicates that for foam, gel, foam gel and temperature-sensitive gel systems, the pressure remains steady or increases slowly during the injection process of the sand filling model, and there is no sudden increase in pressure. These four types of plugging systems all have a good transmission performance, whereas the oil sludge exhibits a poorer performance, which often causes intense pressure fluctuations during the injection process.
2. The experimental evaluation of plugging properties indicates that the order of plugging performance from best to worst is as follows: phenolic resin system > oily sludge system > temperature-sensitive gel system > gel system > foam gel system > foam system. The plugging rate is >90% in the liquid phase of each plugging system.
3. The order of washing resistance of plugging system from largest to smallest is as follows: phenolic resin system > oily sludge system > temperature-sensitive gel system. The plugging rate of the phenolic resin system reaches up to 99.67% after steam flooding at 300 °C. The temperature resistance of the foam and gel system is relatively poor.
4. The effect of plugging a high-permeability channel from largest to smallest is as follows: oily sludge > phenolic resin > gel > temperature-sensitive gel. The recovery rates of the low-permeability sand pipe under 300°C steam flooding are 60%, 46.44%, 44.44% and 37.14%, respectively. The selected plugging systems all have a good plugging effect. After plugging for 300°C steam flooding, the recovery rates of the high-permeability sand pipe are 16.45%, 11.43%, 11.73%, and 3.64%, respectively. Under the experimental conditions, the plugging system blocks the high-permeability layer, which is not completely blocked and retains a certain liquid production capacity.

## Appendix

μ——dynamic viscosity, mPa ⋅ s;
T——Temperature, °C;
PV——Pore volume, Dimensionless;
*P*_*t*_——Breakthrough pressure gradient, MPa ⋅ m^−1^;
*P*_max_——Breakthrough pressure, MPa;
*L*——Length of sand filling pipe, m;
*F*_rrw_——Residual resistance factor, Dimensionless;
*K*_wa_——water test permeability before plugging, mD;
*K*_wb_——water test permeability after plugging, mD;
*η*——plugging rate, Dimensionless.

## Supporting information

S1 File Nature research editing service certification. This document certifies that the manuscript listed below was edited for proper English language, grammar, punctuation, spelling, and overall style by one or more of the highly qualified native English speaking editors at American Journal Experts.
(PDF)

S1 Fig High resolution figures. All Figures of the whole paper along with extra figures are listed.
(PDF)

S1 Table Related data. Data is got from all experiments and used for all of the figures listed in this paper.
(XLSX)
