# Supplementary material for: A high-temperature plugging system for offshore heavy oil thermal recovery
Source: PLoS One. 2018 Jun 22;13(6):e0199709. doi: 10.1371/journal.pone.0199709 (PMC6014645; doi:10.1371/journal.pone.0199709)
Supplement: S1 Fig — All Figures of the whole paper along with extra figures are listed. (PDF) [file pone.0199709.s002.pdf]

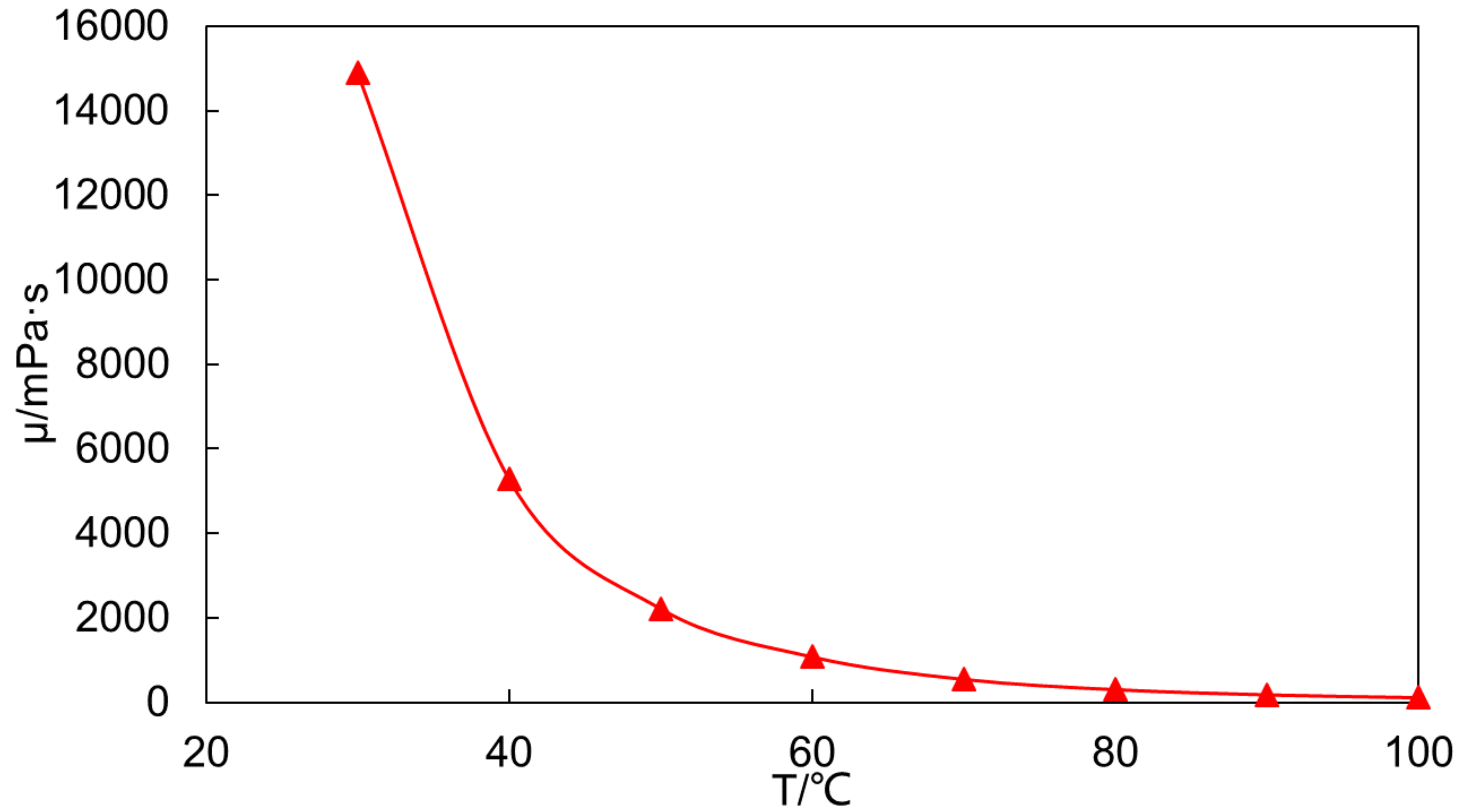

Figure 1 Viscosity-temperature curve of dehydrated crude oil from a heavy oil reservoir at Bohai Sea area

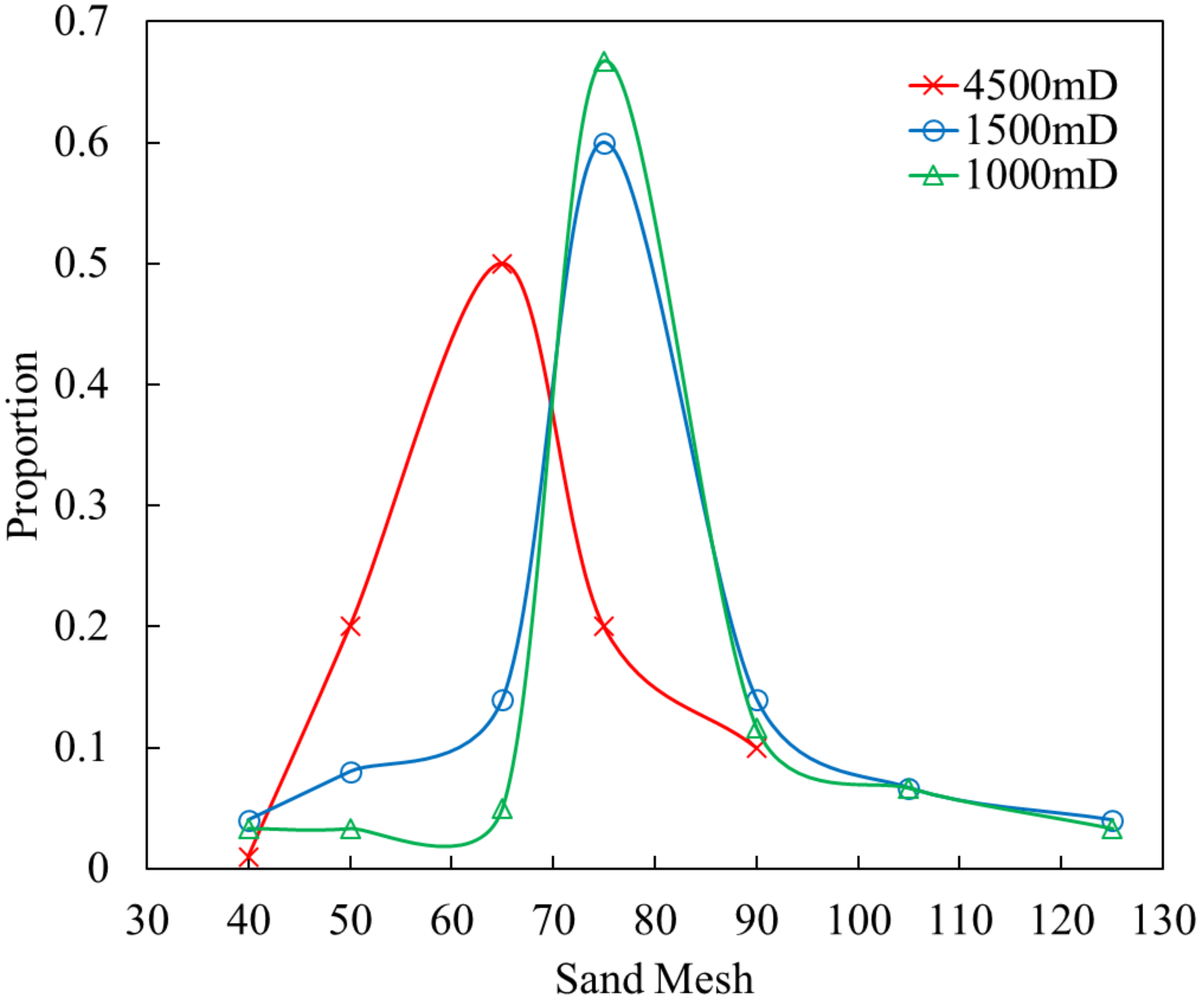

Figure 2 Sand particle diameter distribution curves of artificial cores with different permeability

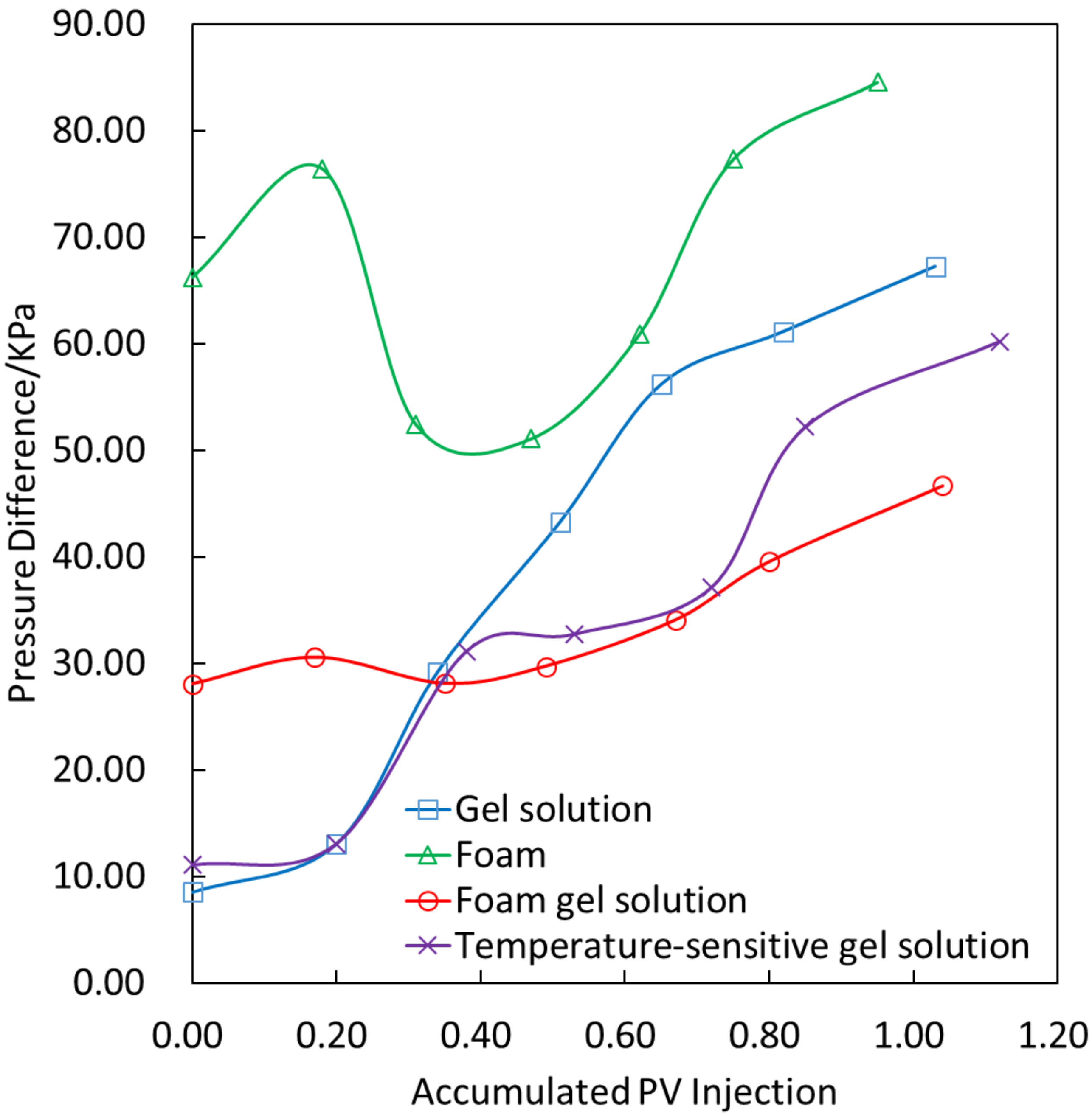
90.00
80.00
70.00
60.00
50.00
40.00
30.00
20.00
10.00
0.00
Pressure Difference/KPa
0.00
0.20
0.40
0.60
0.80
1.00
1.20
Accumulated PV Injection
Gel solution
Foam
Foam gel solution
Temperature-sensitive gel solution

(a) 50℃

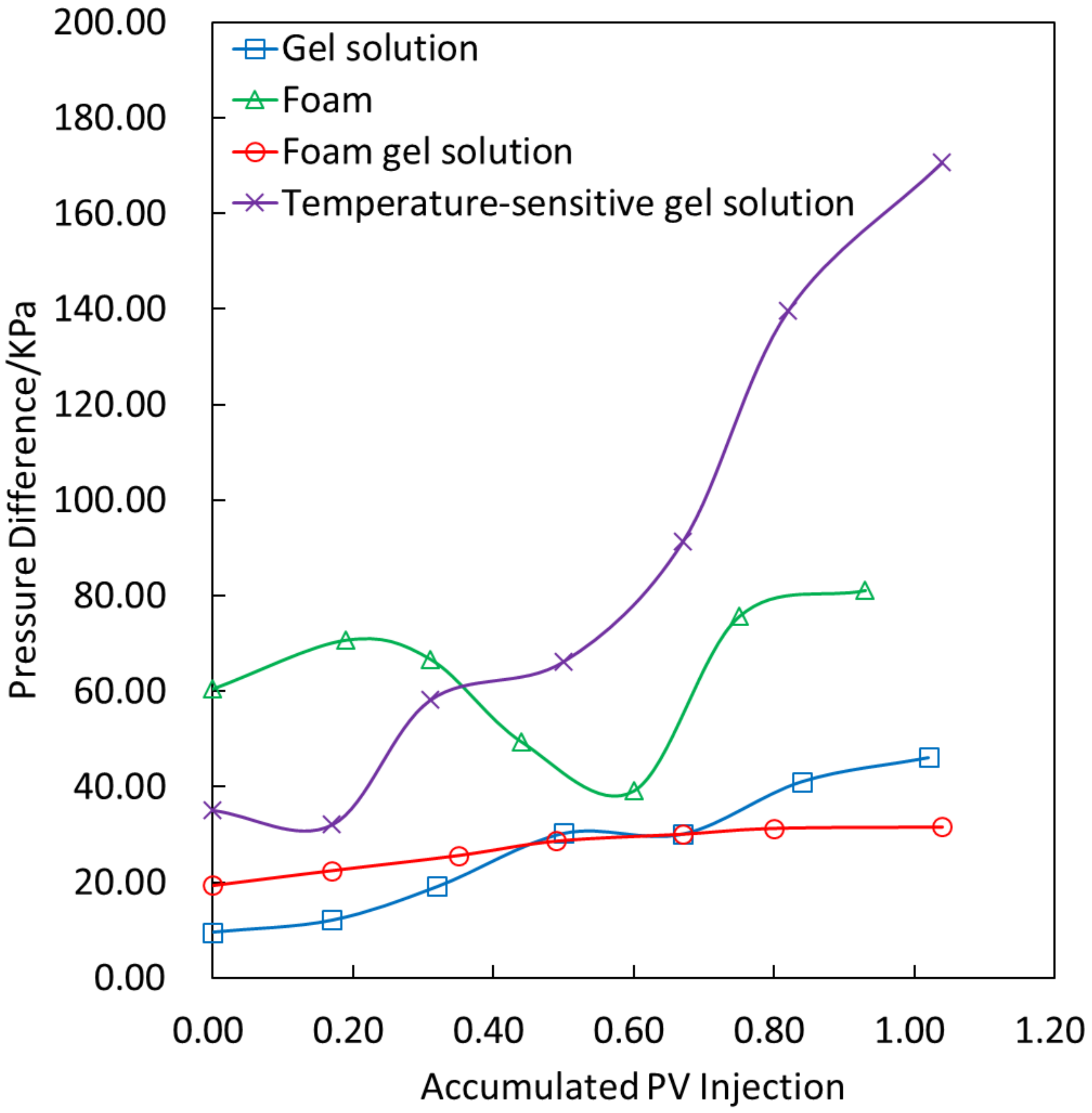

(b) 80℃

Figure 3 Transmission experiments of different plugging systems at 50℃ and 80℃

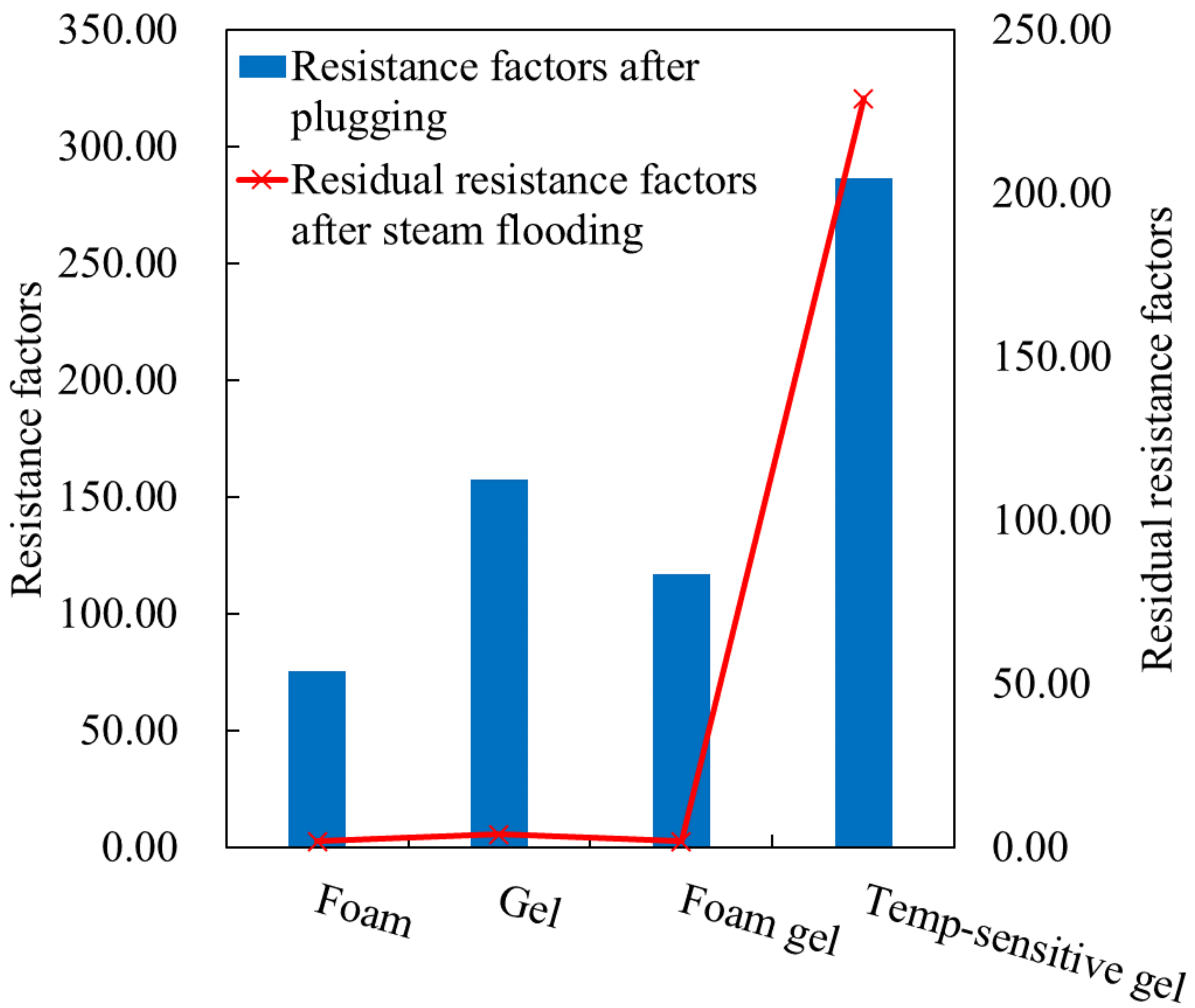
350.00
300.00
250.00
200.00
150.00
100.00
50.00
0.00
Resistance factors
250.00
200.00
150.00
100.00
50.00
0.00
Residual resistance factors
Resistance factors after plugging
Residual resistance factors after steam flooding
Foam
Gel
Foam gel
Temp-sensitive gel

(a) 100℃

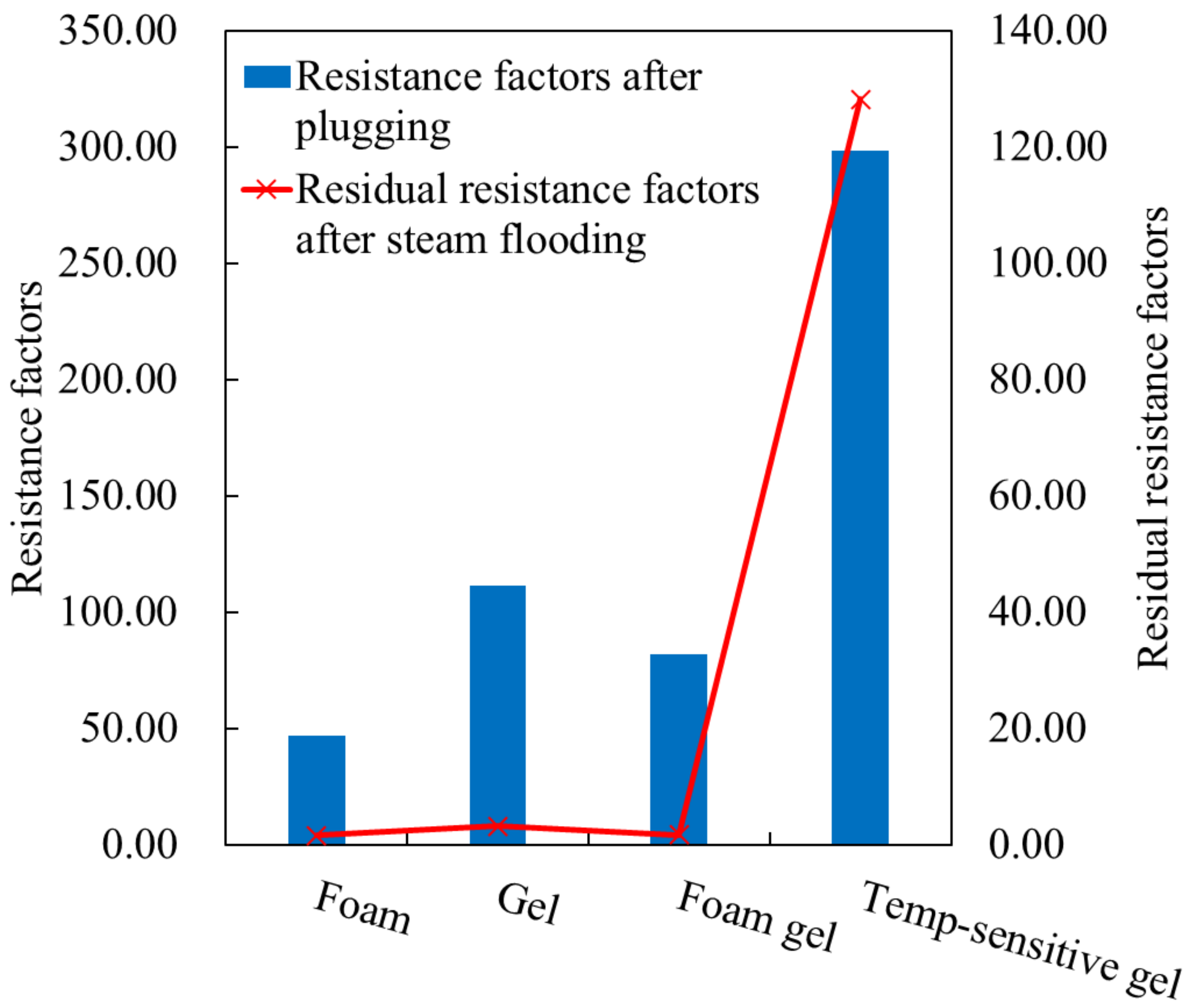

(b) 200℃

Figure 4 Resistance factors after plugging and residual resistance factors after steam flooding of different plugging agents at 100℃ and 200℃

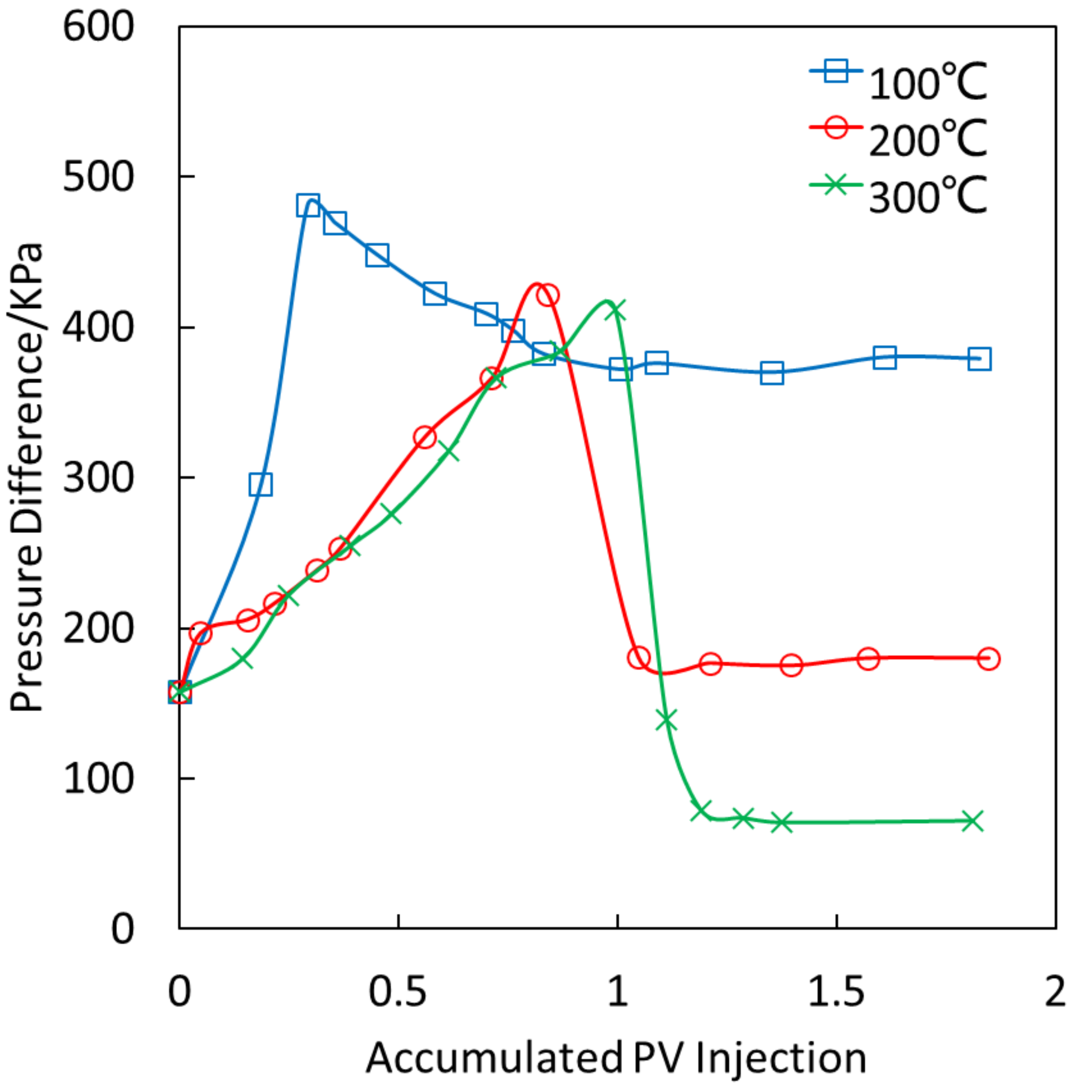

(a) temperature-sensitive gels

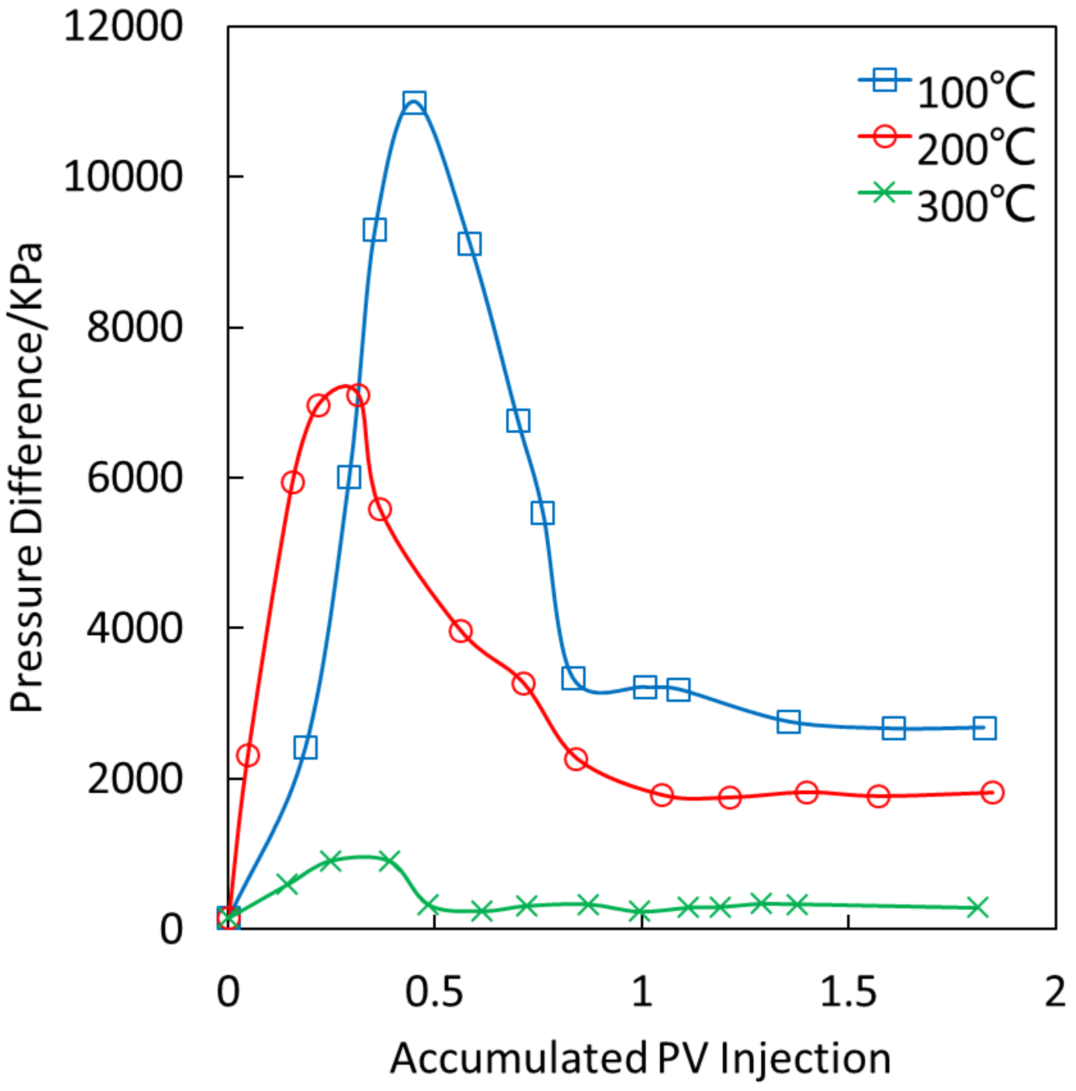

(b) oily sludge

Figure 5 Relations between steam flooding pressure difference and the accumulated injection volume of temperature-sensitive gels and oily sludge after plugging at different temperatures

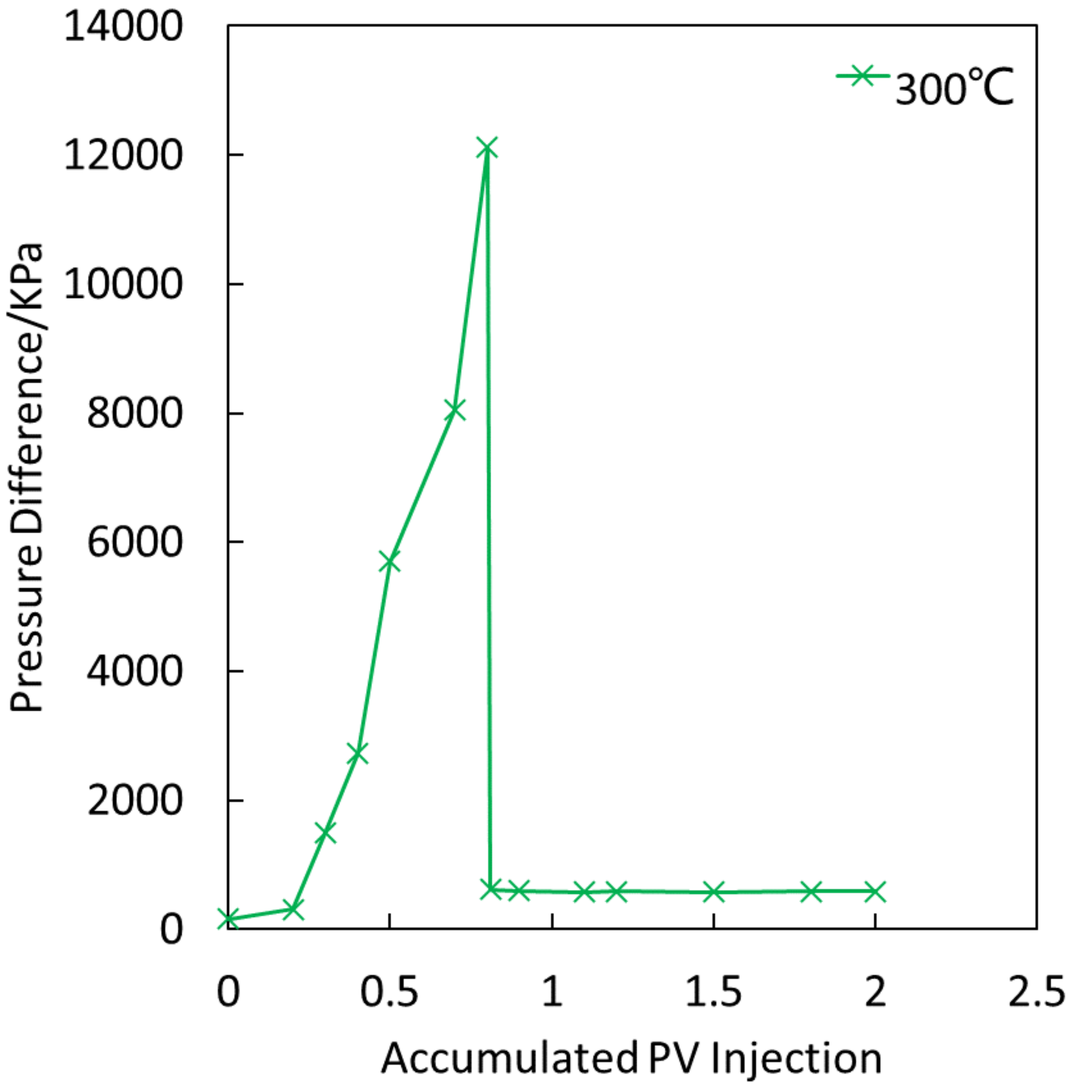

Figure 6 Relations between steam flooding pressure difference and the accumulated injection volume of phenolic resin after plugging at 300℃

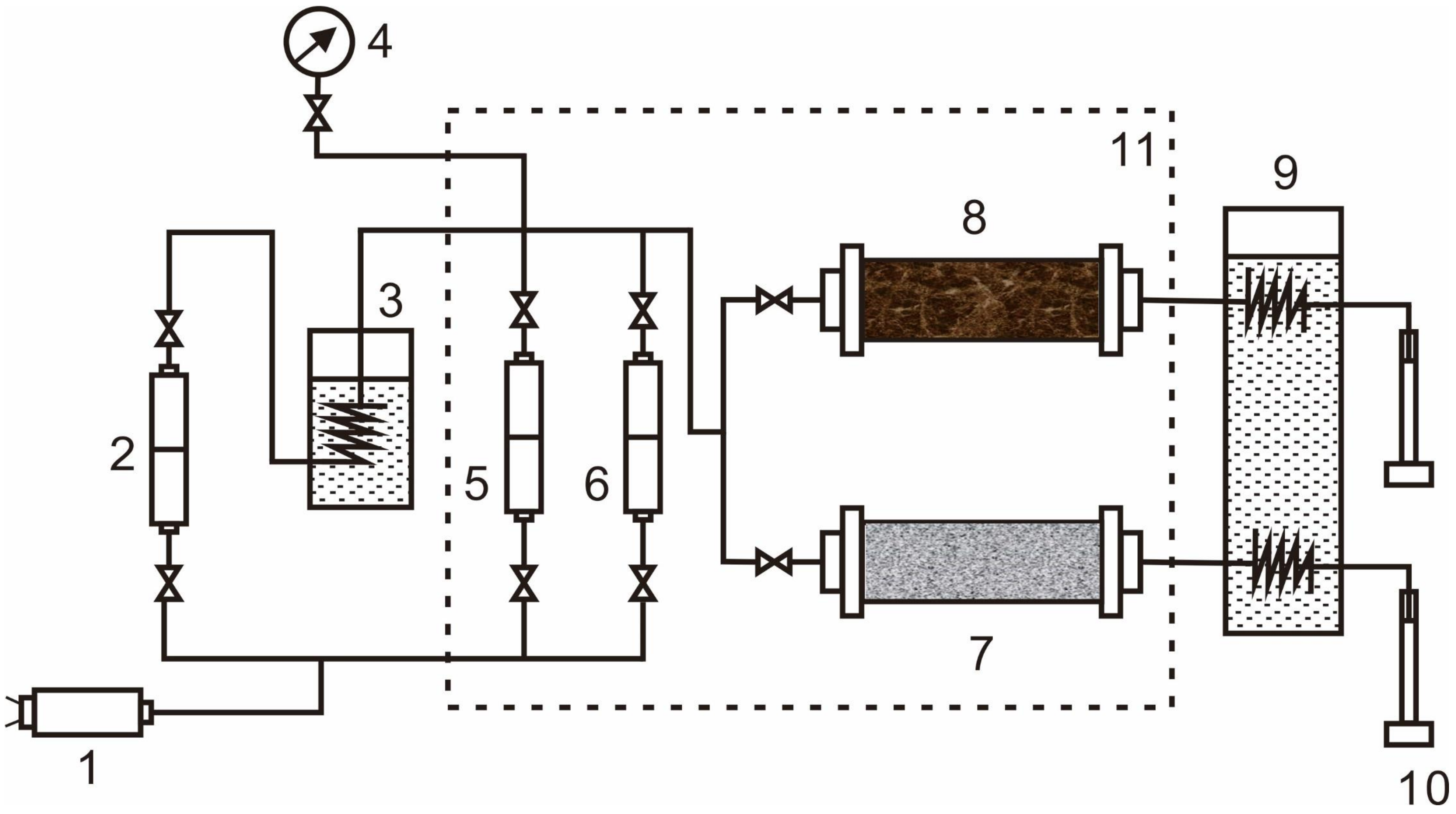

1-constant flow pump; 2-water containers; 3-steam generators; 4-pressure sensors; 5-oil containers; 6-plugging system containers; 7, 8-sand filling pipes; 9-cooling water bath; 10-measuring cylinder; 11-thermostat

Figure 7 Flow diagram of double-tube parallel plugging simulation experiment

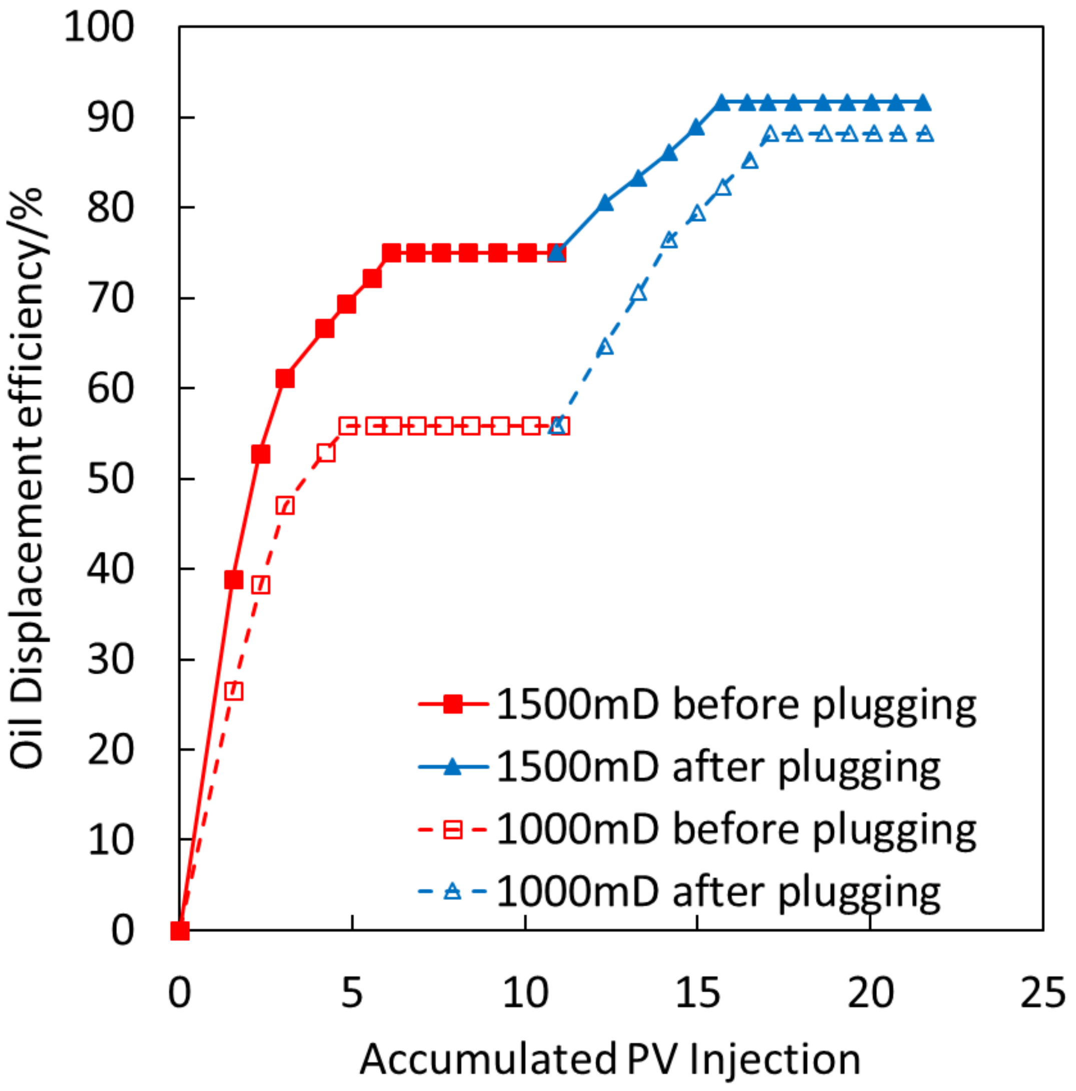
Oil Displacement efficiency/%
100
90
80
70
60
50
40
30
20
10
0
0
5
10
15
20
25
Accumulated PV Injection
1500mD before plugging
1500mD after plugging
1000mD before plugging
1000mD after plugging

(a) 200℃

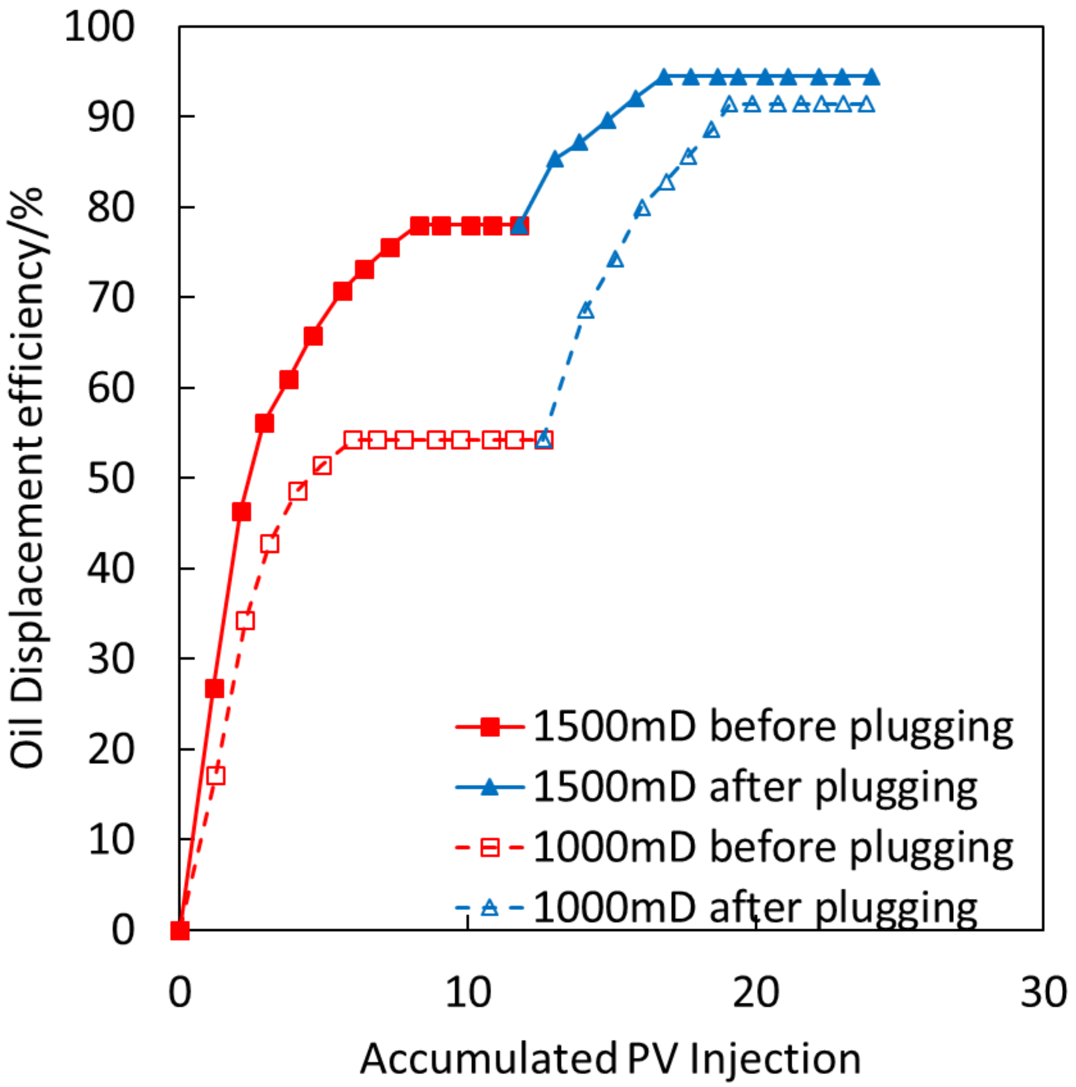

(b) 300℃

Figure 8 Curves of oil displacement efficiency comparison before and after gel plugging at 200℃ and 300℃

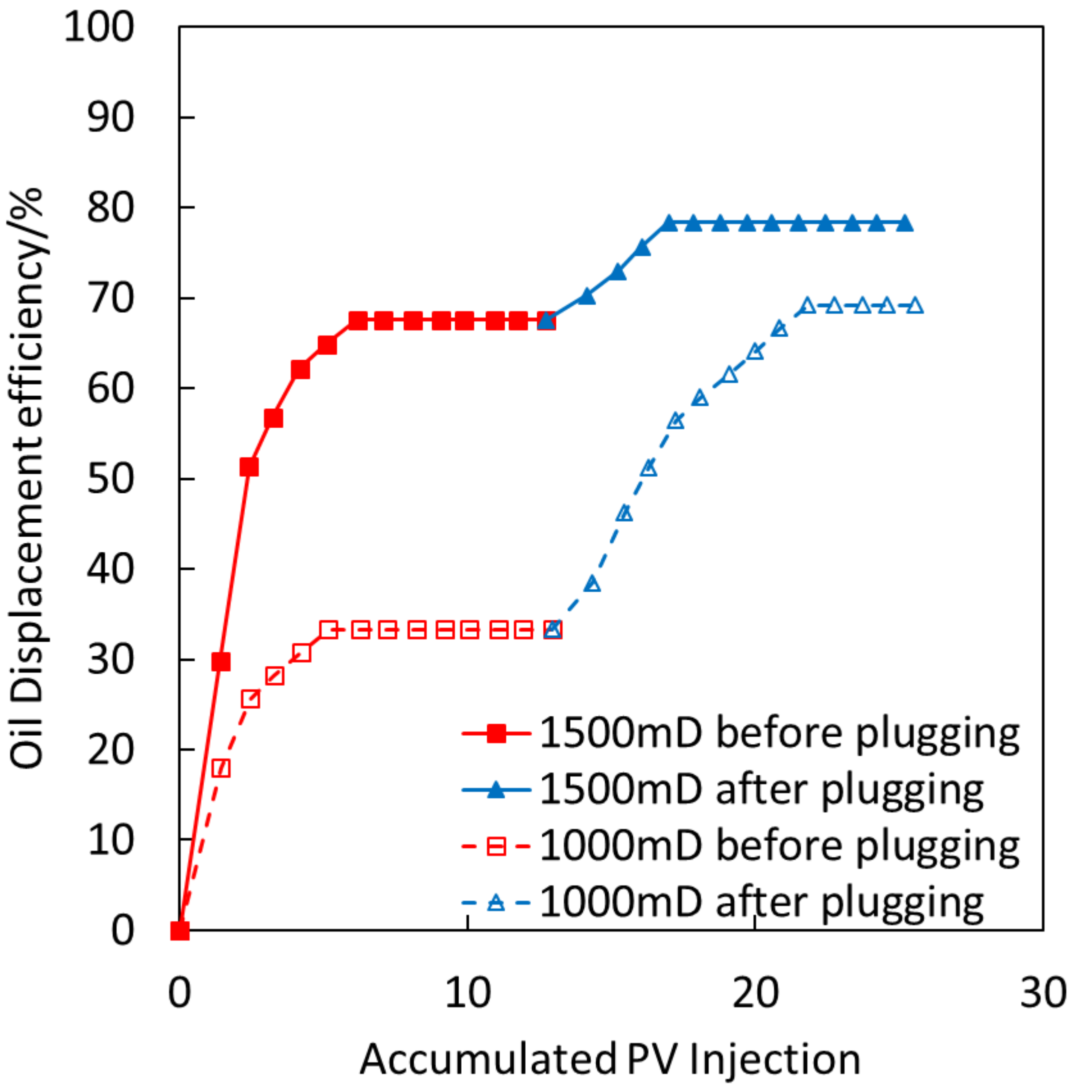

100
90
80
70
60
50
40
30
20
10
0
Oil Displacement efficiency/%
0
10
20
30
Accumulated PV Injection
1500mD before plugging
1500mD after plugging
1000mD before plugging
1000mD after plugging

(a) 200℃

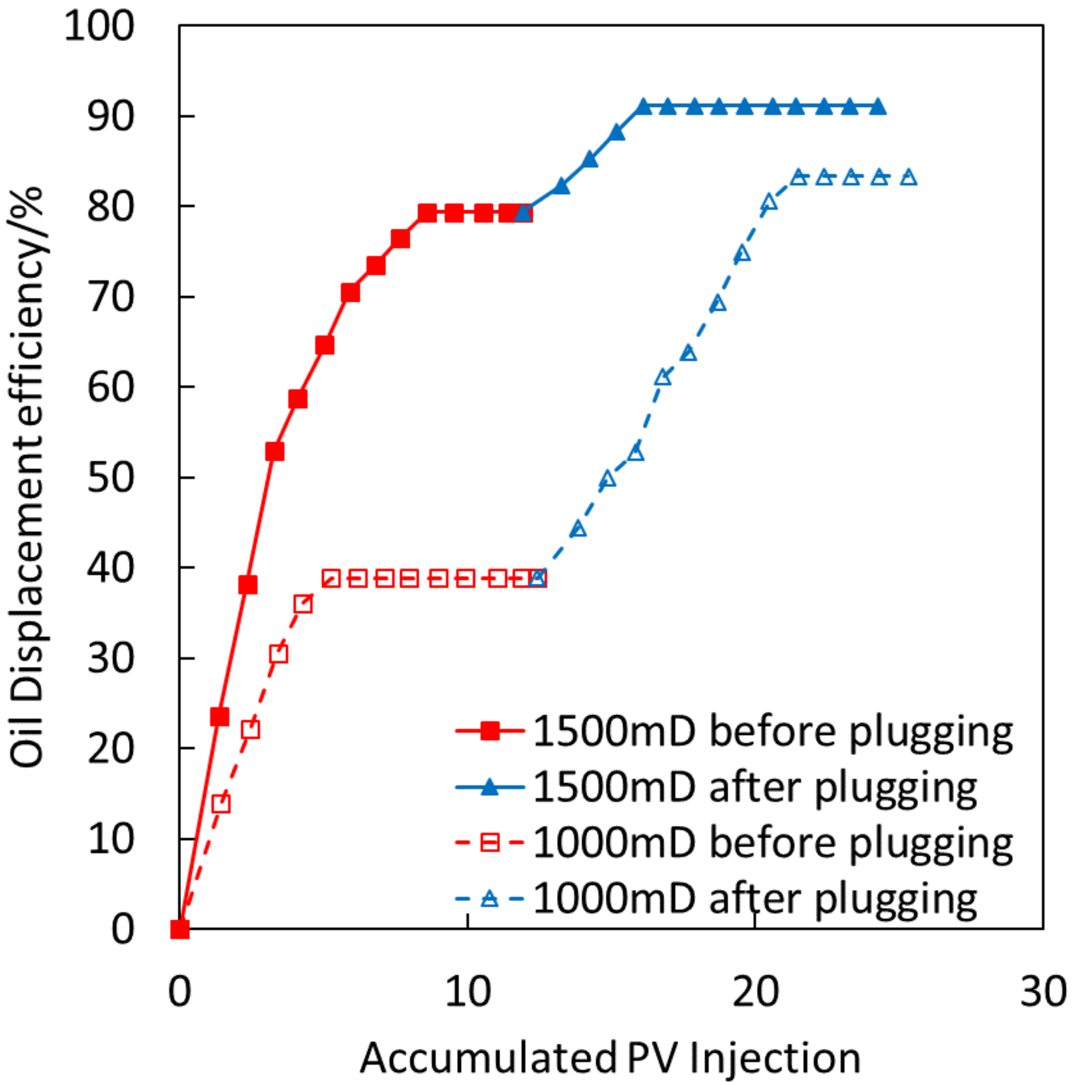

(b) 300℃

Figure 9 Curves of oil displacement efficiency comparison before and after temperature-sensitive gel plugging at 200℃ and 300℃

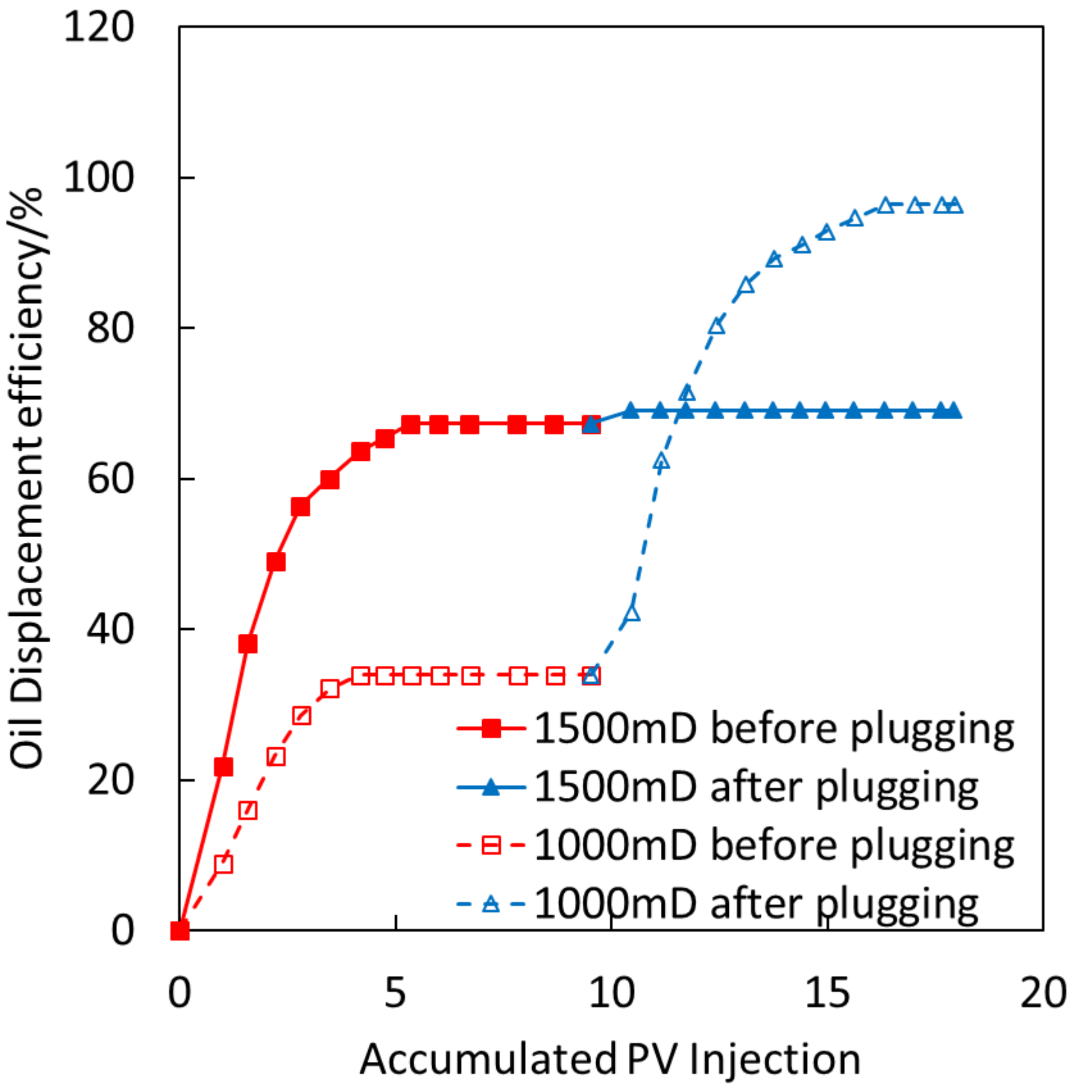

120
100
80
60
40
20
0
Oil Displacement efficiency/%
0
5
10
15
20
Accumulated PV Injection
1500mD before plugging
1500mD after plugging
1000mD before plugging
1000mD after plugging

(a) 200℃

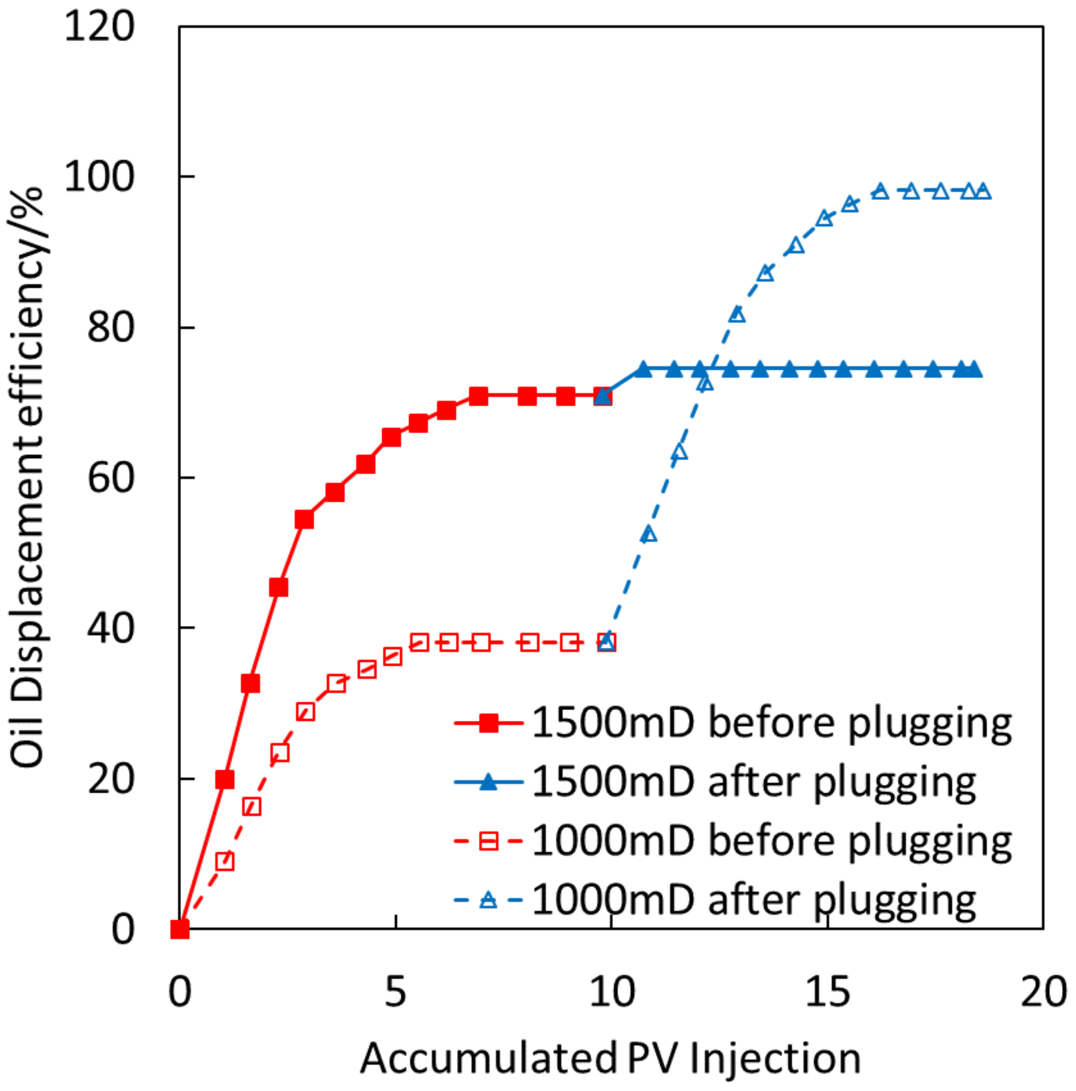

(b) 300℃

Figure 10 Curves of oil displacement efficiency comparison before and after oily sludge plugging at 200℃ and 300℃

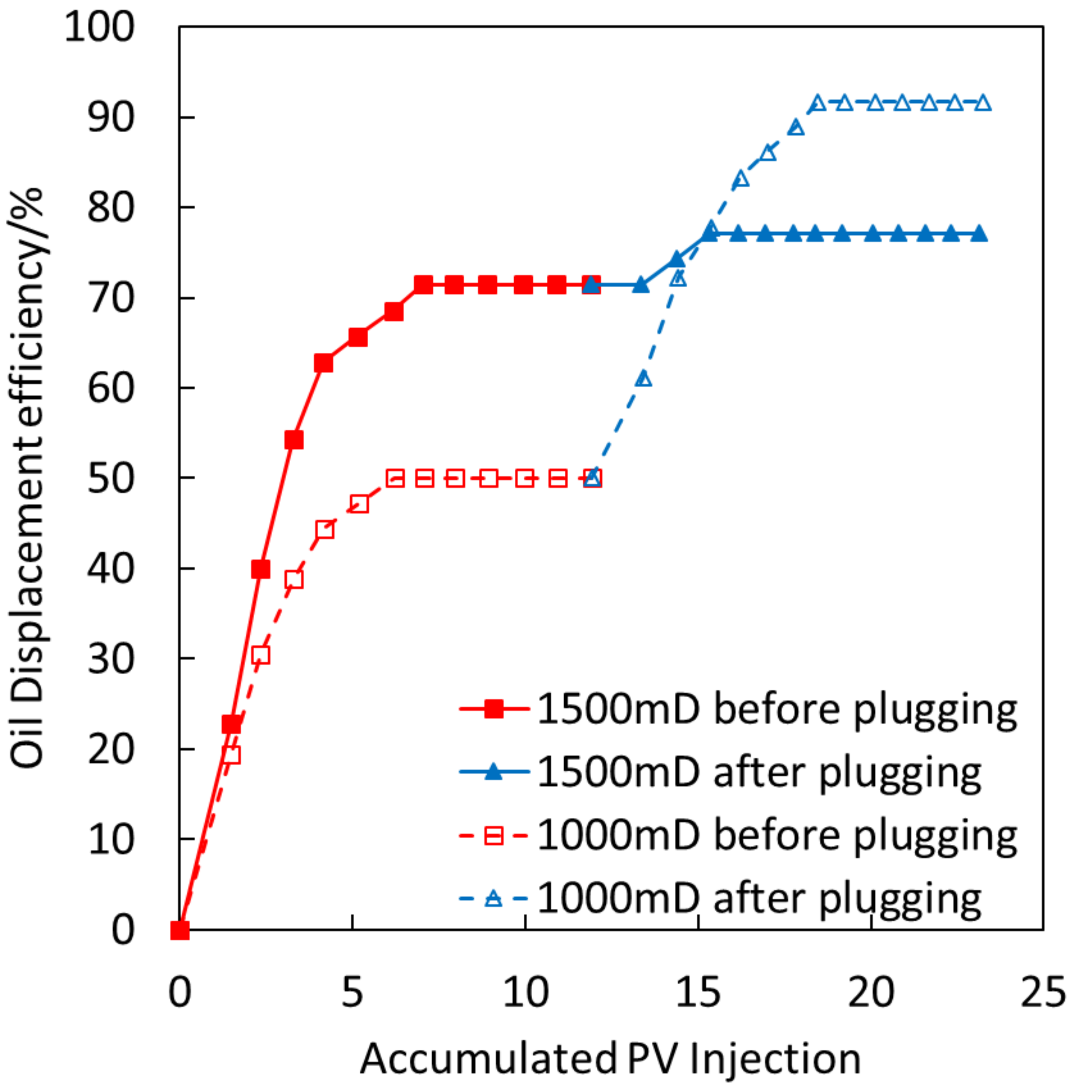

100
90
80
70
60
50
40
30
20
10
0
Oil Displacement efficiency/%
0
5
10
15
20
25
Accumulated PV Injection
1500mD before plugging
1500mD after plugging
1000mD before plugging
1000mD after plugging

(a) 200℃

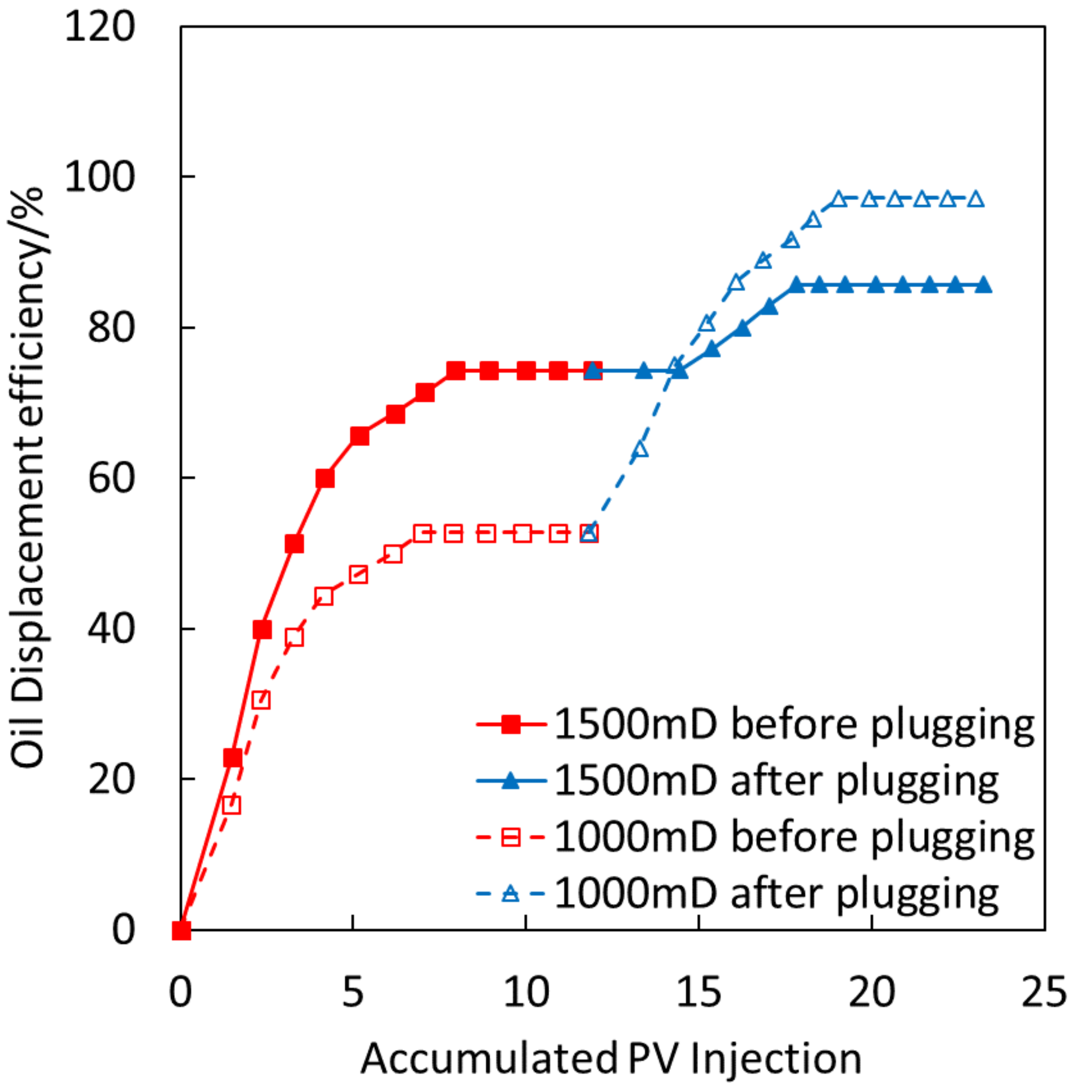

(b) 300℃

Figure 11 Curves of oil displacement efficiency comparison before and after phenolic resin plugging at 200℃ and 300℃

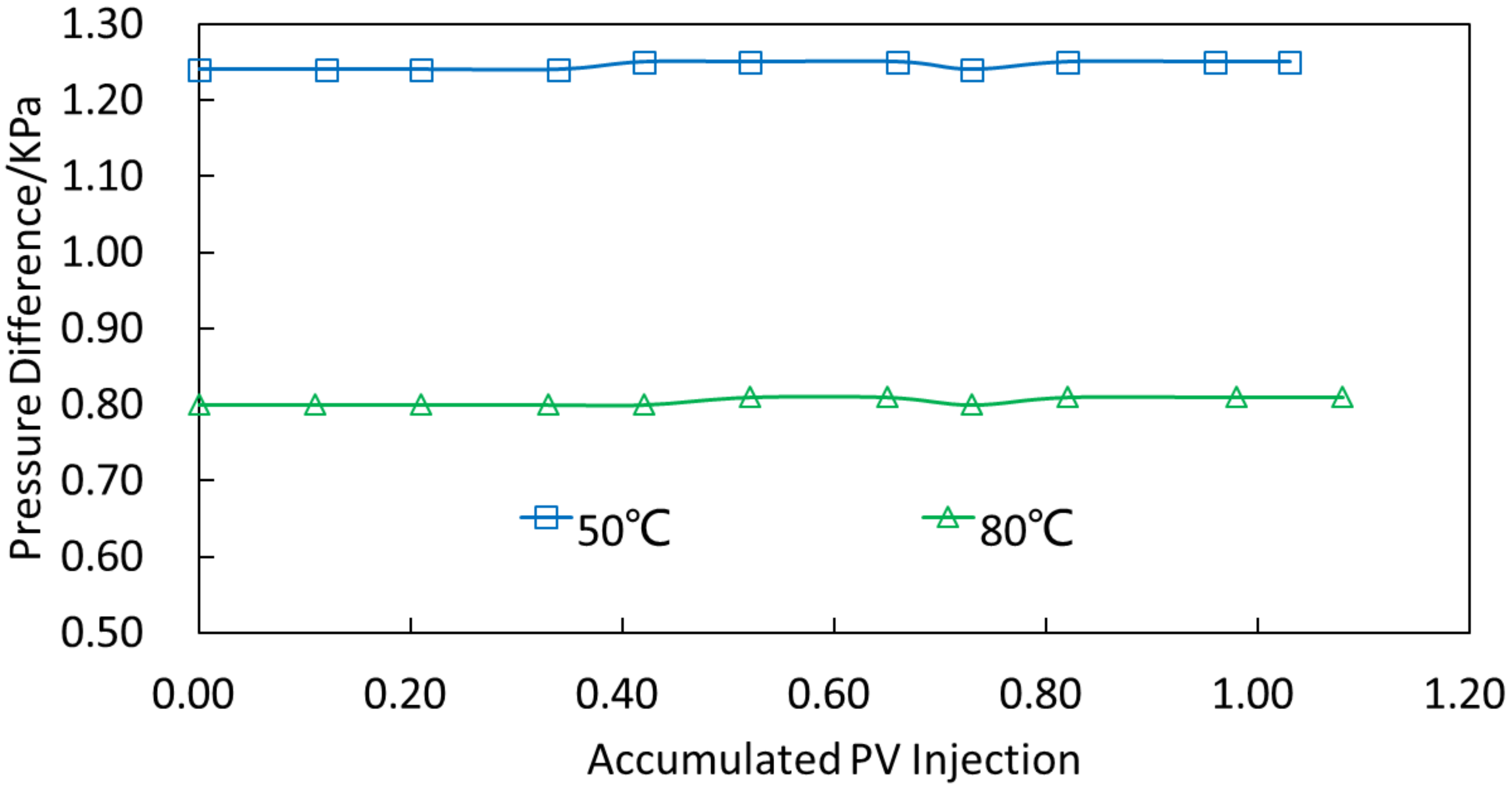

Extra Figure Transmission experiments of phenolic resin solution at 50°C and 80°C
